# Evaluating the impact of improvements in urban green space on older adults’ physical activity and wellbeing: protocol for a natural experimental study

**DOI:** 10.1186/s12889-018-5812-z

**Published:** 2018-07-27

**Authors:** Jack S. Benton, Jamie Anderson, Sarah Cotterill, Matthew Dennis, Sarah J. Lindley, David P. French

**Affiliations:** 1 Manchester Centre for Health Psychology, Division of Psychology & Mental Health, School of Health Sciences, University of Manchester, Coupland 1 Building, Oxford Road, Manchester, M13 9PL UK; 2 Urban Institute, Department of Geography, School of Environment, Education and Development, University of Manchester, Manchester, UK; 3 Centre for Biostatistics, Division of Population Health, Health Services Research & Primary Care, School of Health Sciences, University of Manchester, Manchester, UK; 4 Department of Geography, School of Education, Environment and Development, University of Manchester, Manchester, UK

**Keywords:** Physical activity, Wellbeing, Urban green space, Older adults, Natural experiment, Protocol

## Abstract

**Background:**

Creating or improving urban green space has the potential to be an effective, sustainable and far-reaching way to increase physical activity and improve other aspects of wellbeing in the population. However, there is a dearth of well-conducted natural experimental studies examining the causal effect of changing urban green space on physical activity and wellbeing. This is especially true in older adults and in the United Kingdom. This paper describes a natural experimental study to evaluate the effect of four small-scale urban street greening interventions on older adults’ physical activity and wellbeing over a 1-year period, relative to eight matched comparison sites. All sites are located in deprived urban neighbourhoods in Greater Manchester, United Kingdom.

**Methods:**

Components of the interventions include tree and flower planting, and artificial tree decorations. Eight unimproved comparison sites were selected based on a systematic process of matching using several known objective and subjective environmental correlates of physical activity in older adults. The outcome measures are physical activity and two other behavioural indicators of wellbeing (Connect: connecting with other people; and Take Notice: taking notice of the environment), collected using a newly developed observation tool. The primary outcome is Take Notice behaviour due to largest effects on this behaviour being anticipated from improvements in the aesthetic quality of green space at the intervention sites. Baseline data collection occurred in September 2017 before the interventions were installed in November 2017. Follow-up data collection will be repeated in February/ March 2018 (6 months) and September 2018 (12 months).

**Discussion:**

The present study permits a rare opportunity to evaluate the causal effects of small-scale changes in urban green space in an understudied population and setting. Although the interventions are expected to have small effects on the outcomes, the present study contributes to developing natural experiment methodology in this field by addressing key methodological weaknesses causing high risk of bias in previous natural experimental studies. Key improvements to reduce risk of bias in the present study are rigorous matching of multiple comparison sites and appropriate statistical control of key confounders.

**Trial registration:**

Retrospectively registered with study ID NCT03575923. Date of registration: 3 July 2018.

**Electronic supplementary material:**

The online version of this article (10.1186/s12889-018-5812-z) contains supplementary material, which is available to authorized users.

## Background

Engaging in regular physical activity provides many physical, social and psychological health benefits for older adults [1, 2]. Despite this, physical activity levels tend to decline as adults become older [3, 4]. The rapidly increasing number of older adults (≥ 60 years old) worldwide, due to increased life expectancy, emphasises the need to improve the health of older adults to extend quality of life [5].

Interventions targeted at the individual level to increase physical activity in adults yield modest improvements and often lack long-term effectiveness, and are thus not cost-effective [6] and this lack of long-term effectiveness is also evident in older adults [7]. In contrast, creating a supportive environment at the population level may be a more effective, sustainable and far-reaching approach to increase older adults’ physical activity levels by targeting the wider determinants of health [8]. Providing a physical environment that supports physical activity may be particularly important for older adults because they are likely to spend more time in their local living environment [9].

Many studies have now shown an association between the built environment (e.g. street design, land-use mix, aesthetic qualities) and physical activity levels in the adult [10] and older adult population [11]. One type of built environment that is particularly promising to improve population levels of physical activity is urban green space [12]; defined as “all publicly owned and publicly accessible open space with a high degree of cover by vegetation, e.g. parks, woodlands, nature areas and other green space” ([13], p. 110) within urban areas. However, there is a lack of evidence on the effects of urban green space specifically in older adults [14, 15] and findings from this small evidence base in older adults are mixed [16]. The effects of urban green space on older adults may be different to the effects on younger adults because many of the known moderators of the relationship between urban green space and physical activity are strongly linked to age, such as usage of urban green space, physical activity preferences, health and mobility [12, 17].

The quality of the evidence to date is limited in a number of ways. First, most of the evidence on urban green space and physical activity is cross-sectional, which limits our ability to infer causality. To infer causality, evaluations of natural experiments have become a priority in this field [15, 18, 19]. Natural experiments are ‘real world’ events or changes that cannot be manipulated or controlled by the researcher and divides a population into exposed and unexposed groups [20]. Natural experimental studies can provide stronger inferences about causality than cross-sectional studies due to the temporal order of exposure (change to environment) and outcome (physical activity). Only a handful of studies have investigated causal effects of changes to urban green space using natural experiments: two recent systematic reviews of studies evaluating the effect of the built environment on physical activity [11] and active travel [21] in older adults found only one natural experimental study.

Second, most research on urban green space has been conducted in the United States (US) and Australia, and there is a lack of studies in the United Kingdom (UK) and elsewhere in Europe [11, 19]. There are many differences between the US and UK in terms of key variables that affect physical activity levels, including differences in climate, population density, transportation networks and physical activity patterns [22, 23]. These differences make it difficult to generalise findings from the US to Europe.

Third, the methodological rigour of the small number of natural experimental studies evaluating the effect of the built environment on physical activity in adult populations is weak. A recent review by Benton et al. [24] evaluated the risk of bias in natural experiments that have evaluated the effects of changing the built environment on physical activity. They identified 12 natural experimental studies (15 physical activity outcomes) on the basis of having strong experimental designs from three existing systematic reviews; nine of these studies evaluated urban green space interventions. They found that all outcomes had an overall critical (*n* = 12) or serious (*n* = 3) risk of bias, thus suggesting that the strongest studies conducted to date are weak, a conclusion in line with other recent systematic reviews in this area that included risk of bias assessments [15, 25, 26].

Benton et al. provided eight recommendations to improve the rigour of future natural experimental studies in this field, based on Medical Research Council (MRC) guidance for using natural experiments [20] as well as other relevant literature (e.g. [15]); the present study is designed to implement these recommendations:

1. Publishing study protocols with a priori analyses specified;
2. Better matching of comparison sites and more nuanced use of graded exposure;
3. Use of multiple comparison sites;
4. Sample size calculations;
5. Use of adequate outcome measurements;
6. Measuring exposure to the intervention at the individual level;
7. Better reporting of samples and interventions; and;
8. Controlling for confounding domains (in statistical analyses).

Urban green space may improve health and wellbeing by several mechanisms, not just physical activity [18]. There has been increasing attention on the effect of urban green space on a wider set of wellbeing indicators [18, 27, 28]. For instance, urban green space that is a positive sensory and symbolic resource (e.g. visual and audible information of value to users) may encourage people to access and draw upon that space more often, providing a pleasant setting for activities such as social interaction and mindful cognitions [27, 29]. It is therefore important to understand how urban green space influences other indicators of wellbeing, in addition to physical activity.

In common with physical activity research, there is a scarcity of evidence on the causal effects of urban green space on wellbeing (i.e. natural experimental studies) [30]. Studies have also often relied on unvalidated measures of wellbeing [31]. However, the science of urban wellbeing is in its infancy. In particular, issues in defining wellbeing may, in part, explain the lack of rigorous studies on the effect of urban green space on wellbeing [18].

An objective method of measuring wellbeing is to measure associated behavioural indicators of wellbeing. On behalf of the UK Government’s Foresight project, New Economics Foundation (NEF) conducted a review of the wellbeing literature and identified behaviours for which there was good evidence that engaging in these behaviours improved an individual’s sustained wellbeing. These behaviours are collectively known as the Five Ways to Wellbeing [32]: Be Active (engage in physical activity); Connect (connect with others); Take Notice (awareness of the environment); Keep Learning (learn new activities); Give (give back to the community). Since there is evidence that these five behaviours are linked to improved wellbeing [32], each of these five behaviours can be used as proxy measures of wellbeing.

A recent natural experimental study used direct observations of behaviour (systematic observation) to measure three of the Five Ways behaviours (Be Active, Connect, Take Notice) that are relevant to use of open spaces. They were measured before and after improvements to an urban green space site in central Manchester, UK; which included installation of shade-tolerant planting, an inner-city lawn and vegetation management [27]. The researchers observed significant increases in usage and wellbeing behaviours in the intervention site at 1-year follow-up compared to a matched comparison site. Therefore, systematic observation of behavioural indicators of wellbeing offers a feasible method of objectively measuring and quantifying wellbeing in the context of the environment.

### Research aim and objectives

The overall aim of this natural experimental study is to investigate the effect of planned changes in urban green space on older adults’ physical activity and wellbeing. The specific objectives are as follows:

1. To examine whether small-scale urban green space interventions increase older adults’ Take Notice behaviour in comparison to matched comparison sites where no such changes occur (primary outcome). The primary outcome is Take Notice behaviour due to largest effects on this behaviour being anticipated from improvements in the aesthetic quality of green space at the intervention sites;
2. To examine whether small-scale urban green space interventions increase the number of older adults’ using the intervention sites in comparison to matched comparison sites where no such changes occur (secondary outcome);
3. To examine whether small-scale urban green space interventions increase older adults’ physical activity levels (sedentary, walking, vigorous activity) or Connect behaviour in comparison to matched comparison sites where no such changes occur (exploratory outcomes).

## Feasibility study

### Background

To inform the main natural experimental study, a feasibility study was carried out in July 2017. The specific objectives of the feasibility study were to determine: (1) how many days of observation per week and hours per day are needed to provide a valid estimate of older adults’ activity in a UK urban setting; (2) what times of the day should observations be carried out to capture variation in older adults’ activity across the course of a day; and (3) any key differences in older adults’ activity patterns on weekdays compared to weekends.

The feasibility study employed MOHAWk (Method for Observing pHysical Activity and Wellbeing) [33]: a newly developed tool for systematically observing physical activity and two other behavioural indicators of wellbeing in small urban green spaces such as pocket parks, tree-lined streets and green corridors along waterways (see Methods in the Main Study for further description of the tool).

### Methods

Observations were carried out by two observers using MOHAWk at the same time at two separate sites in Greater Manchester (GM). Observations were conducted 8 am-6 pm in 50-min observation periods (e.g. 8–8.50 am, 9–9.50 am etc.) by one observer for seven consecutive days from Saturday to Friday in Intervention Site 1 (Table 1, Fig. 6). At the same time, a second observer conducted observations in a different site for five consecutive days from Monday to Friday.

### Results

To estimate the average reliability of overall daily counts of older adults, single rater, two-way random effects, consistency measure intraclass correlation coefficients (ICC) were used. ICCs were calculated for all possible abbreviated schedules: combinations of 2, 3, or 4 days per week compared to the full 5 days per week (weekdays only). It was found that, on average, observing on 2 days a week can produce consistency approaching that obtained by observing 5 days a week (ICC = .82).

Similarly, ICCs were used to estimate the average reliability of hourly counts of older adults for the following abbreviated schedules: combinations of 2, 3, or 4 h per day compared to the full 10 h per day. Two hours a day collection was defined as 1 h in the first half of the day (8 am-1 pm) and 1 h in the second half of the day (1-6 pm); three times a day was defined as morning (8 am-12 pm), early afternoon (12-3 pm) and late afternoon/ early evening (3-6 pm); and four times a day was defined as early morning (8-10 am), late morning (10 am-12 pm), early afternoon (12-3 pm) and late afternoon/ early evening (3-6 pm). It was found that, on average, observing 4 h per day can produce consistency approaching that obtained by observing 10 h per day (ICC = .86).

**Fig. 1:**
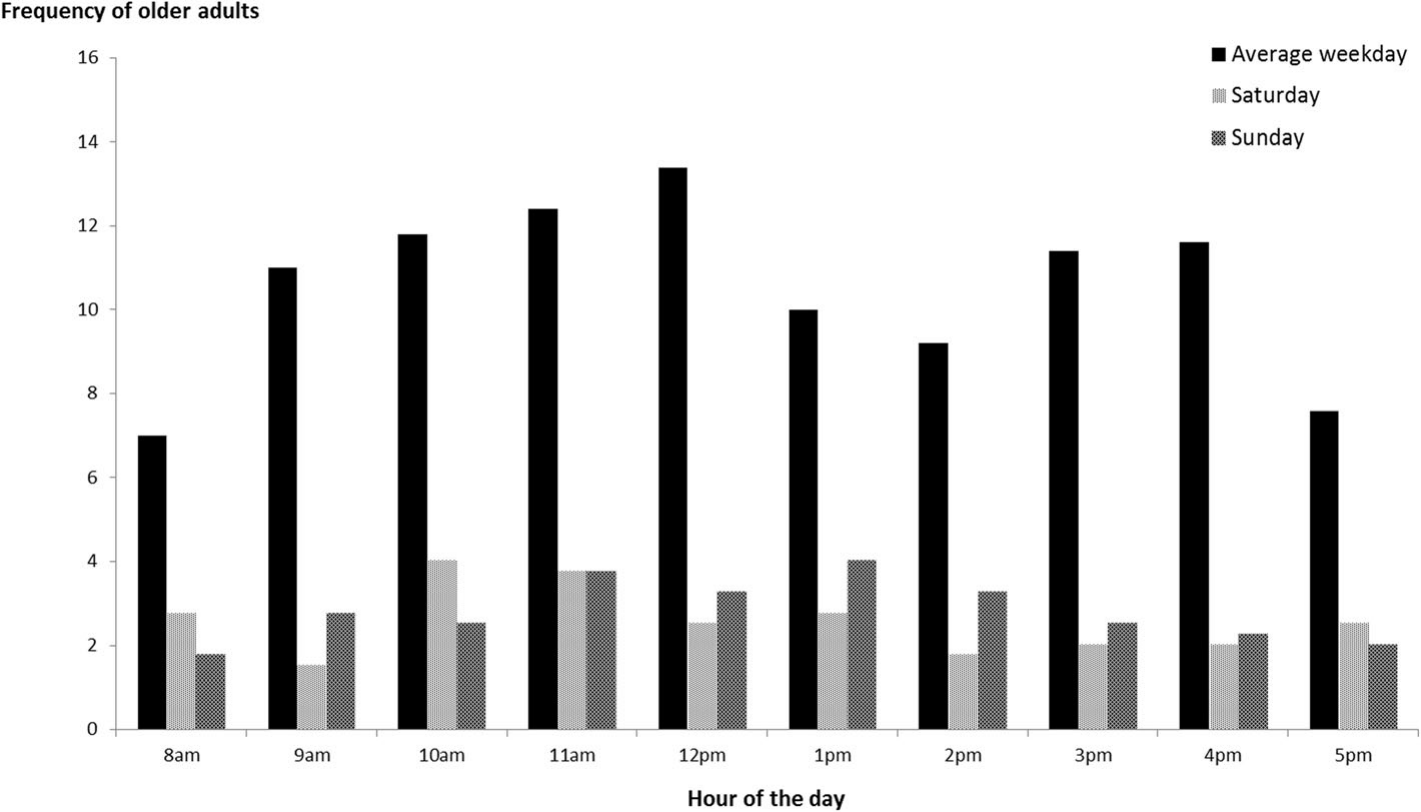
Frequency of older adults in Intervention site 1 during the feasibility study. Counts of older adults were observed between 8 -6 pm on weekdays (average of Monday to Friday), Saturday and Sunday during the feasibility study in July 2017

In general, counts of older adults were higher in the morning (8 am-12 pm) than early afternoon (12-3 pm) or late afternoon/ early evening (3-6 pm) (Fig. 1). This is in line with previous studies suggesting that that older adults are generally more active in the mornings, particularly in the late morning, rather than afternoons and evenings [34–36], with very little activity occurring beyond 6 pm [35]. As shown in Fig. 1, there were fewer older adults observed on weekend days than weekdays.

### Discussion

The observation data for older adults in a UK urban setting informed decisions about the frequency and timing of observations in the main study.

## Methods (main study)

### Study design

This is a prospective controlled before and after natural experimental study of the effects of changes in urban green space, with four intervention and eight matched comparison sites in GM: two comparison sites per intervention site.

### Study population

Data will be collected on all individuals (infants, children, teens, adults, older adults) entering the target area during observations. However, this study is focused on older adults and therefore primary analyses will only consider data from older adults. Secondary analyses will consider data from adults.

### Procedure

#### Comparison site matching

Due to the absence of randomisation in natural experiments, comparison (or control) groups ought to be matched on all important variables that influence the outcome to strengthen internal validity and improve accuracy of the estimated intervention effect [20]. However, previous studies in this area have often used poorly matched comparison groups; in particular, an absence of any matching based on objective features of the environment that correlate with physical activity e.g. population density, street connectivity [24]. To address this issue, the present study selected comparison sites that were matched to corresponding intervention sites, using several key objective and subjective environmental variables. Two comparison sites were matched to each intervention site to increase the likelihood of finding comparable comparison sites.

**Fig. 2:**
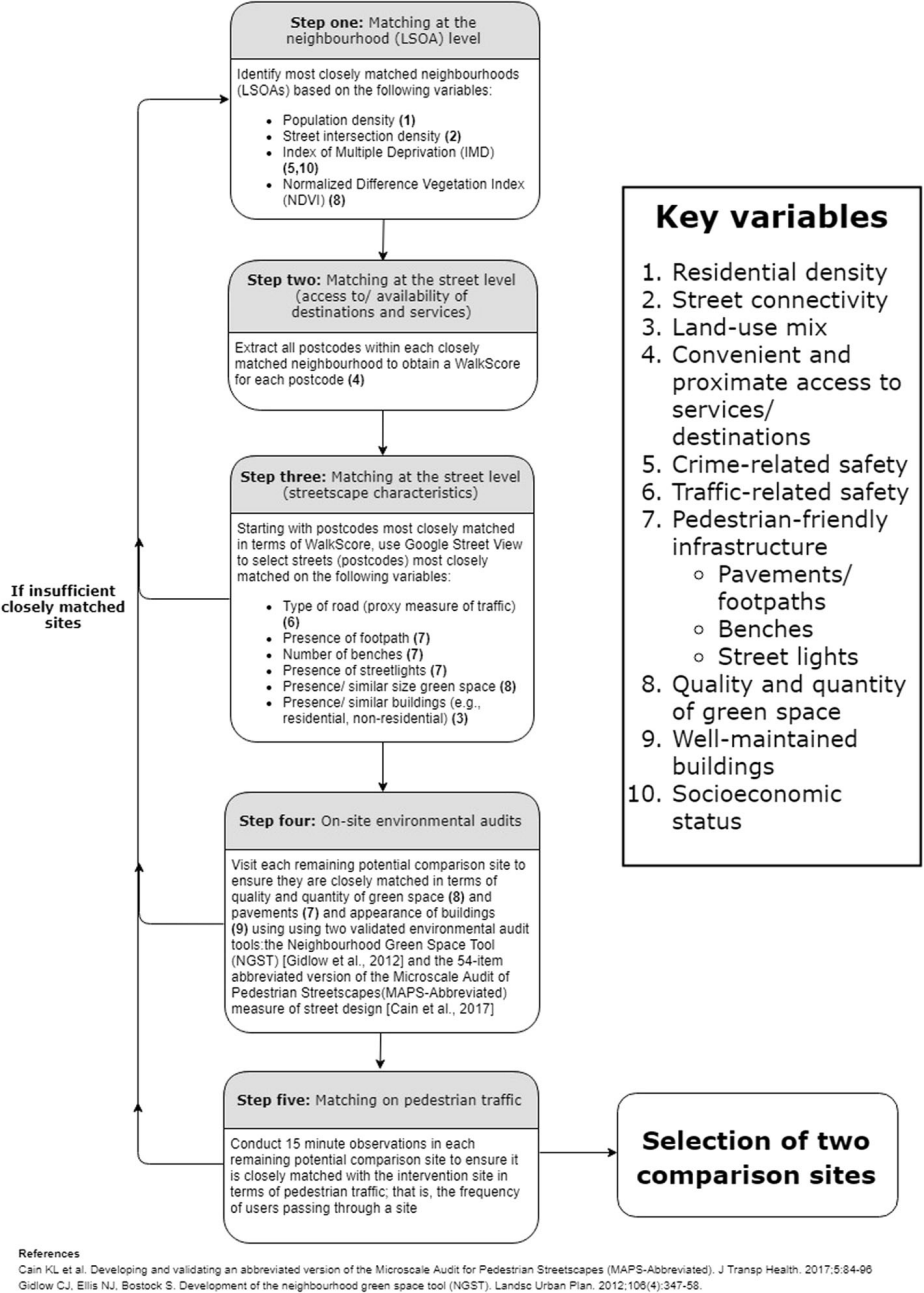
Overview of the five steps constituting the comparison site matching and selection process. Numbers in brackets refer to the key variables used for comparison site matching

There is an absence of evidence on how characteristics of the built environment may influence wellbeing; accordingly, all variables that comparison sites were matched on were from three recent systematic reviews that have investigated built environmental correlates of older adults’ physical activity: one qualitative [37] and two quantitative systematic reviews [11, 21]. Figure 2 displays the variables that were reported as consistent correlates of older adults’ physical activity in at least two of these reviews: these correlates represent the key variables that were used for comparison site matching.

There are no agreed-upon standards for how researchers ought to identify matched comparison sites when studying the effects of the built environment on physical activity [24]; therefore, a five-step process of matching was developed. An overview of this process is displayed in Fig. 2 and each step is described in more detail in Additional file 1.

#### Timing

Baseline data collection was conducted in September 2017 before any changes in urban green space occurred in November 2017. Follow-up will be conducted at two time points: February/ March 2018 (6 months) and September 2018 (12 months). The first follow-up is intended to measure initial short-term effects of the interventions, 6 months post-baseline and 3 months after completion of the interventions. The second follow-up will be conducted 1 year after baseline, at the same time of year to control for seasonal variation.

#### Observation schedule and procedure

Informed by data from the feasibility study, observations will be conducted over 2 days, four times a day (weekdays only) at each time point, resulting in a total of eight observation periods for each site at each time point. Observations will be conducted at four set observation periods per day: morning (10-11 am), lunchtime (12-1 pm), afternoon (3-4 pm), and evening (5-6 pm). These times were found to capture the biggest variation in older adults’ activity across the day (Fig. 1), whilst also providing sufficient time for breaks and possible travel to other sites in between observation periods.

**Fig. 3:**
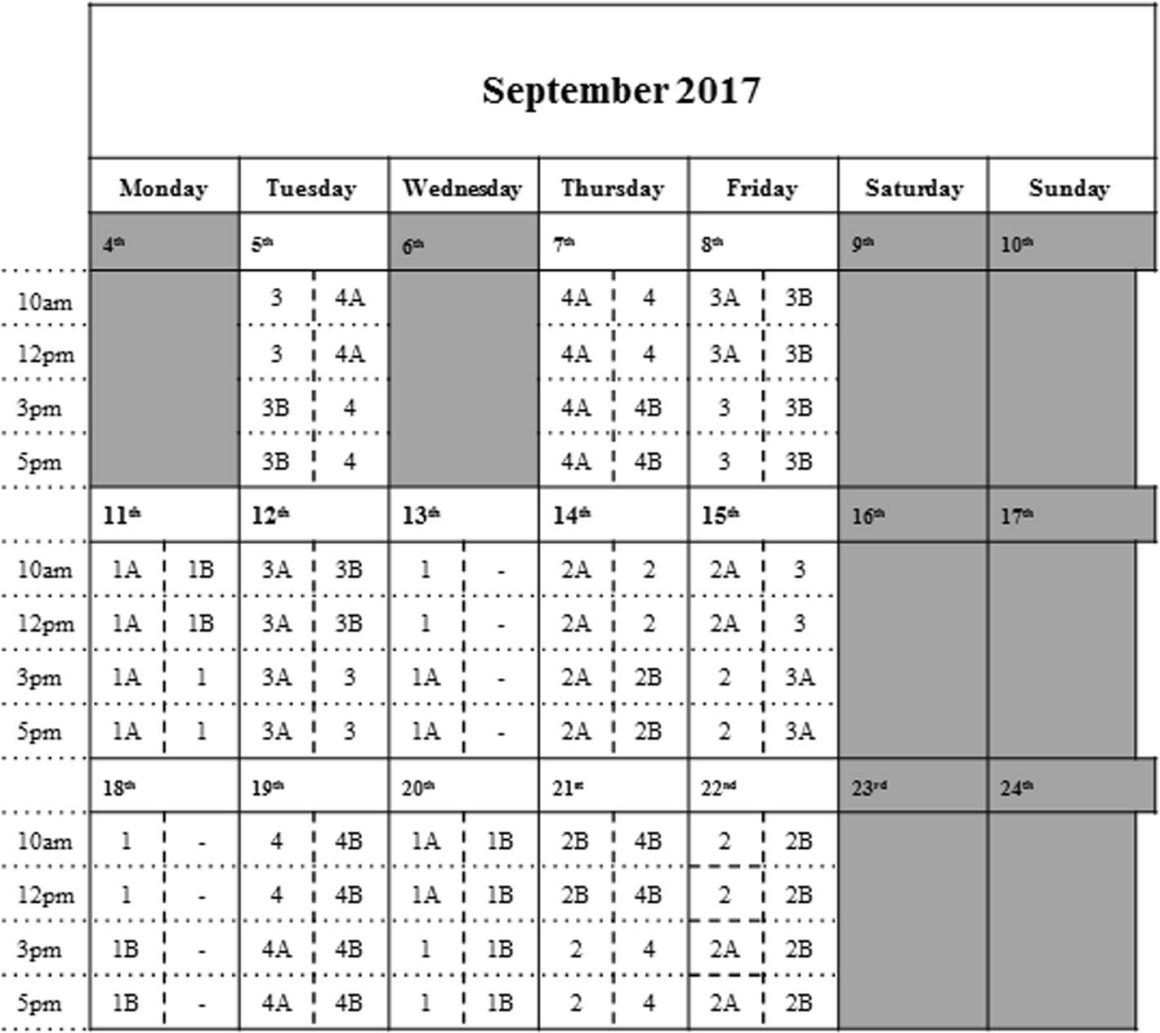
Observation schedule for two observers during baseline data collection for all intervention and comparison sites. The two columns within each day correspond to the two observers i.e. one column for each observer. The numbers/ letters refer to intervention and comparison sites e.g. ‘1’ is Intervention site 1, and ‘1A’ and ‘1B’ are the corresponding comparison sites: Comparison site 1A and Comparison site 1B, respectively. See Table 1 for details on each intervention and comparison site

Observations for each intervention site and the two corresponding comparison sites will be spread over 2 weeks. This will provide a more robust assessment of activity over a longer period rather than observing activity during a single week. Observations will be counterbalanced to control for week, day of week and time of day. The observation schedule used for baseline data collection is displayed in Fig. 3; this schedule will be replicated at all follow-ups. Any missed observations (e.g. due to illness) will be rescheduled for the same day of the next available week.

The same procedure will be used as set out in the MOHAWk observation manual (Additional file 2). All observers will be trained in using MOHAWk and will be required to demonstrate high agreement with the trainer before making observations in the present study. Prior to observations, observers will visit each site to agree on the boundaries of the target area in which all participants will be recorded. Target areas are of similar size between corresponding intervention and comparison sites, and the same target areas will be used at all time points (see Additional file 3).

Data will be recorded using pencil and paper. Data will be entered into SPSS at each time point once data collection is completed.

### Outcome measure: Systematic observation of physical activity and wellbeing

Systematic observation of activity in each of the intervention and comparison sites will be carried out using MOHAWk [33]: an observation tool that measures three levels of physical activity intensity (Sedentary, Walking and Vigorous) and two other behavioural indicators of wellbeing (Connect: connecting with other people; and Take Notice: taking notice of the environment). This tool applies interval time sampling techniques using continuous observation of activities and characteristics of all individuals entering predefined target areas during hour-long observation periods. MOHAWk requires observers to record the following characteristics for all observed participants: age (older adults defined as ≥60 years of age), gender, ethnicity and whether they are overweight or have a disability (require assistance to move). Observers also document weather conditions (precipitation) and incivilities in the target area (e.g. general litter, graffiti). MOHAWk is a newly developed tool, for which there is preliminary evidence of validity [33].

MOHAWk was chosen because existing validated tools for systematic observation of physical activity, such as SOPARC (System for Observing Play and Recreation in Communities) [38], use momentary scans of activity and were developed for outdoor environments that attract consistently high numbers of users or large groups (e.g. large regional parks). The planned study will evaluate small outdoor environments that have lower numbers of users and less consistent usage, thus momentary scans would be unable to reliably capture people’s activity within or passing through that space. MOHAWk also measures objective behavioural indicators of wellbeing; wellbeing has predominantly been measured used self-report, which is more susceptible to recall bias and poor response rates [39].

### Greening interventions

The following descriptions are in line with the Template for Intervention Description and Replication (TIDieR) checklist [40].

This study is set in GM; a large metropolitan county in North West England with a population of around 2.8 million and containing ten metropolitan boroughs. GM is undergoing rapidly increasing urbanisation. As a result, integrating green space into this highly urbanised county is becoming a political priority for GM [41]. GM is therefore a strong case study for evaluating the effects of changes in urban green space on physical activity and wellbeing.

**Fig. 4:**
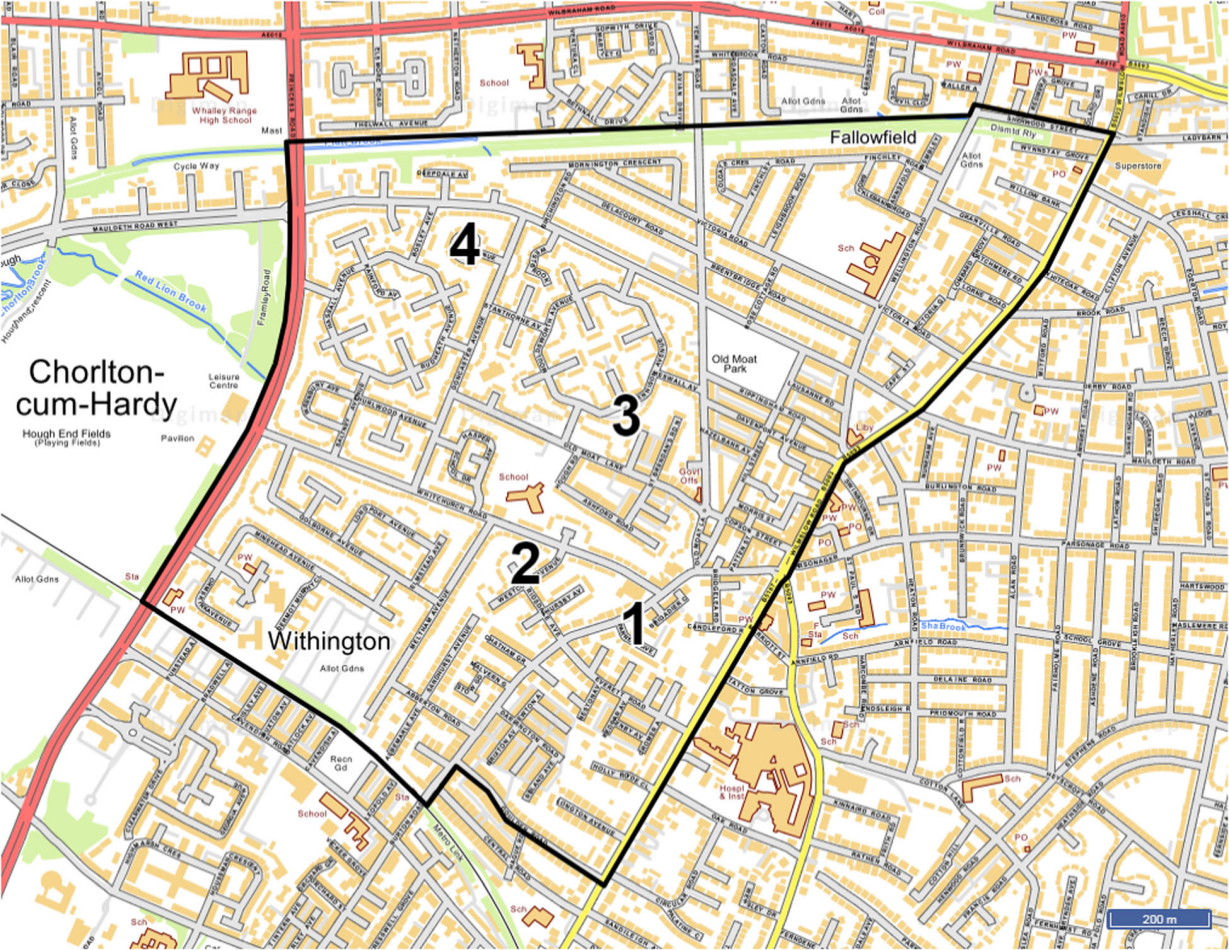
Map showing the boundary of Old Moat and location of all intervention sites. © Crown Copyright and Database Right (2018). Ordnance Survey (Digimap Licence)

This study will evaluate four urban green space interventions designed and implemented by Southway Housing Trust: a housing association in GM. Southway Housing Trust state that the aim of the interventions is to increase the number of people actively using specific areas targeted for environmental improvements and improve wellbeing in the local community (P. Reece, personal communication). The intervention sites are located in Old Moat (Fig. 4): a suburban ward with a population of 14,657 located in the city of Manchester. The population of Old Moat ward is relatively young compared to other wards in Manchester, although there are a high proportion of older adults living in Southway Housing Trust properties [42]. Manchester is ranked as the fifth most deprived local authority in England [43] and Old Moat is ranked as the 22nd most deprived ward in Manchester out of 32 wards [44] (based on the Index of Multiple Deprivation (IMD) Score [45]).

The interventions are located on four publicly accessible sites; the total size of the floor area of green space in each of the intervention sites is small, ranging from 0.09 to 0.35 acres. Components of the interventions include tree and flower planting (expected to bloom by March 2018) and artificial tree decorations such as strings of small electric lights and tree socks (the interventions are hereafter collectively referred to as urban street greening). The total cost for all components across all four intervention sites is approximately £6000, although this excludes artist fees connected to the project. All components of the interventions were implemented within a 1 week period in November 2017 by two arborists, two local artists, staff members at Southway Housing Trust, and local community members from Old Moat and a local school. The Neighbourhood Green Space Tool (NGST) [46], a UK validated environmental audit tool for measuring the quality of green space, will be used to measure the environmental changes at the intervention sites.

**Fig. 5:**
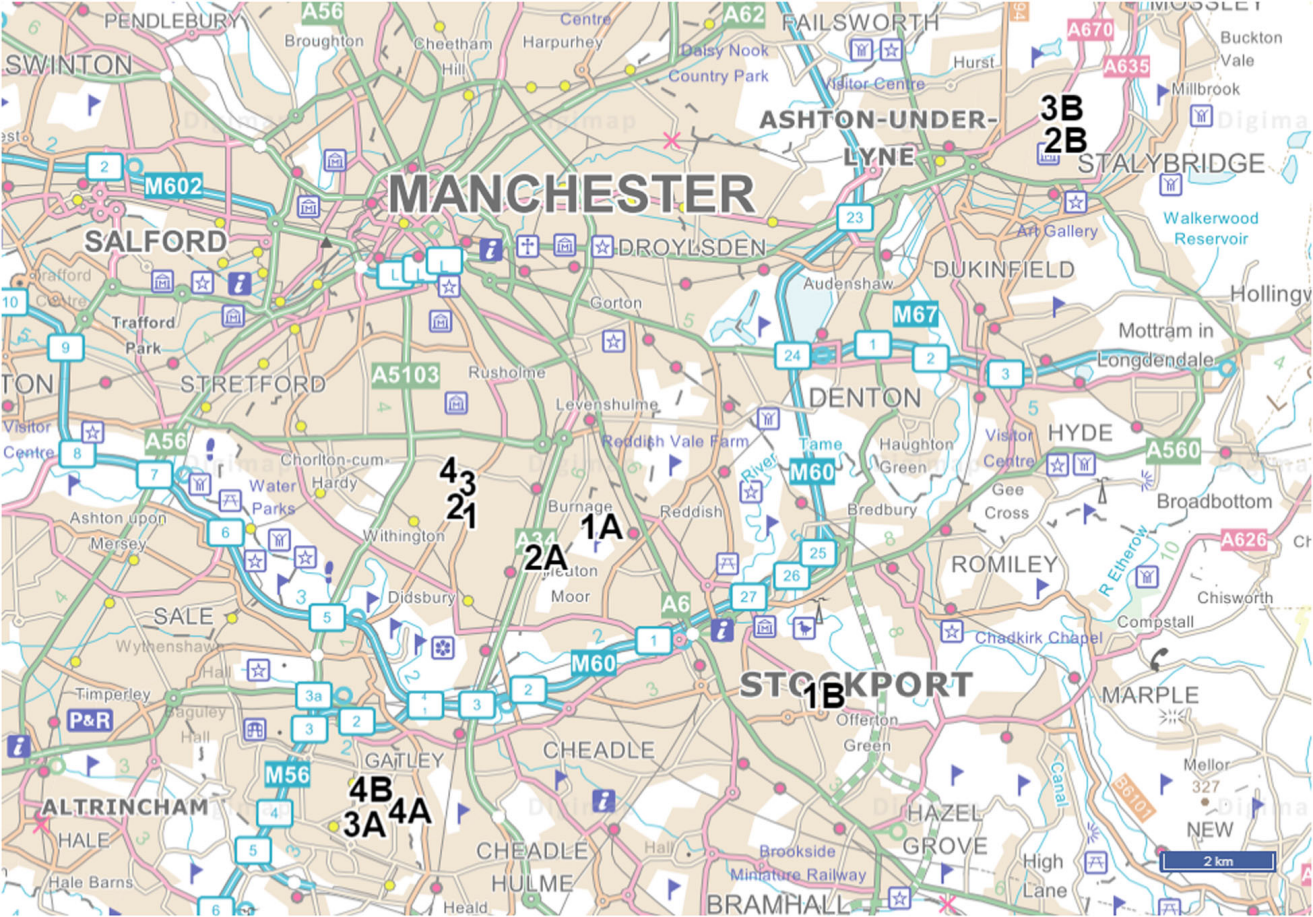
Map showing the location of all intervention and comparison sites in Greater Manchester. The numbers/ letters refer to intervention and comparison sites e.g. ‘1’ is Intervention site 1, and ‘1A’ and ‘1B’ are the corresponding comparison sites: Comparison site 1A and Comparison site 1B, respectively. See Table 1 for details on each intervention and comparison site. © Crown Copyright and Database Right (2018). Ordnance Survey (Digimap Licence)

**Fig. 6:**
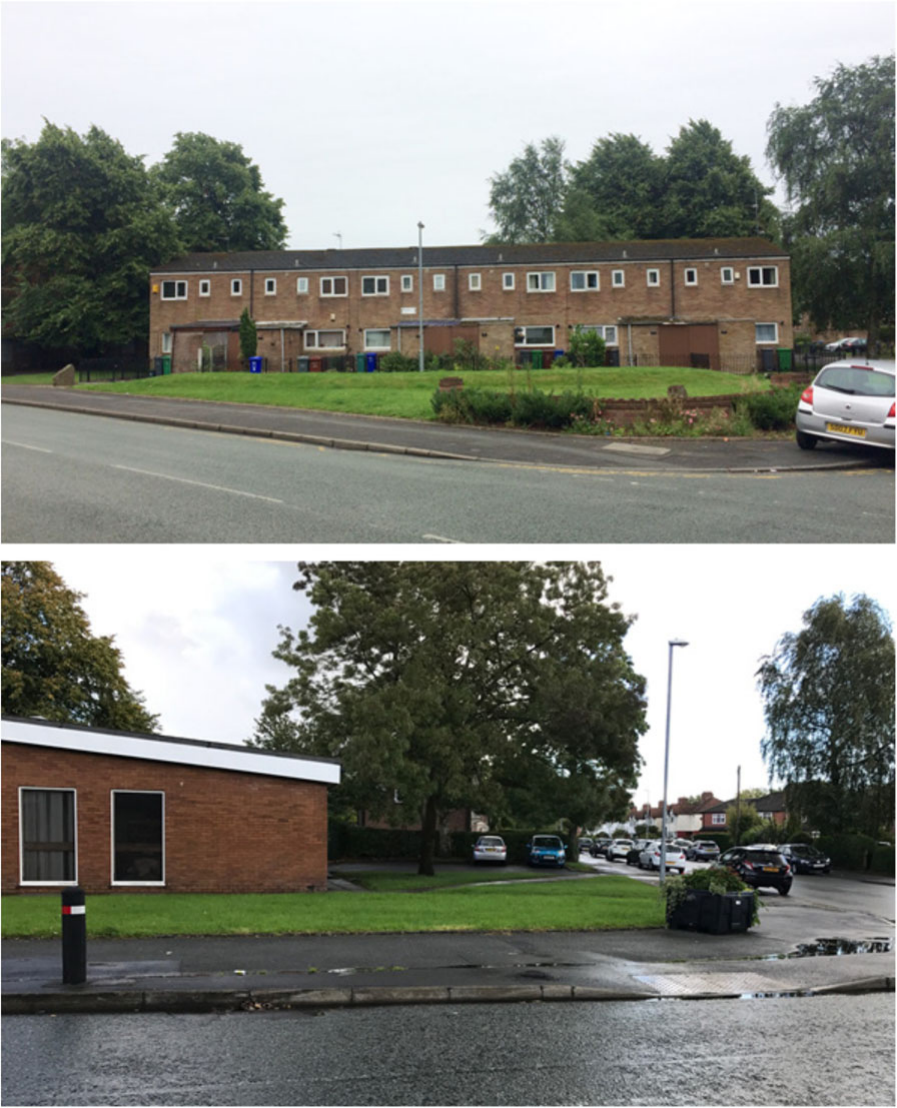
Intervention site 1 (top) and Comparison site 1A (bottom) at baseline. Photographs taken by Jack Benton

A description of the key characteristics and locations of all intervention and comparison sites can be found in Table 1 and Fig. 5 respectively. An example of one of the intervention sites and one of the corresponding comparison sites at baseline are shown in Fig. 6.

**Table 1:** Key characteristics of all intervention and comparison sites

Intervention and comparison sites	Location (postcode)	Intervention components	Floor area of green space (acres)	LSOA^a^	Population density (persons Haˉ^1^)^b^	Intersection density (per 1000mˉ^2^)^c^	IMD^d^	NDVI^e^	WalkScore^f^
Intervention site 1	M20 3GB	2 planted trees; bulb planting	0.09	Manchester 038C	79.50	15.37	23.08	0.38	94
Comparison site 1A	M19 1EN	–	0.05	Manchester 034B	82.58	16.13	29.87	0.47	71
Comparison site 1B	SK2 6DS	–	0.21	Stockport 019D	69.92	16.18	18.02	0.40	63
Intervention site 2	M20 1FU	12 planted trees; bulb planting; artificial tree decorations (string lights)	0.27	Manchester 038A	69.77	15.54	36.84	0.37	90
Comparison site 2A	M20 6FE	–	0.24	Manchester 040A	69.85	14.27	38.24	0.44	83
Comparison site 2B	OL6 8HH	–	0.14	Tameside 004C	73.19	16.68	51.14	0.46	54
Intervention site 3	M20 1GF	3 planted trees; artificial tree decorations (string lights, tree socks)	0.22	Manchester 035A	80.88	15.52	47.92	0.46	92
Comparison site 3A	M22 9PS	–	0.12	Manchester 050D	78.94	17.36	54.98	0.40	80
Comparison site 3B	OL6 8HW	–	0.19	Tameside 004C	73.19	16.68	51.14	0.46	63
Intervention site 4	M20 1AQ	8 planted trees; bulb planting; artificial tree decorations (string lights, tree socks)	0.35	Manchester 035A	80.88	15.52	47.92	0.46	48
Comparison site 4A	M22 9SZ	–	0.17	Manchester 050D	78.94	17.36	54.98	0.40	82
Comparison site 4B	M22 9PU	–	0.13	Manchester 050D	78.94	17.36	54.98	0.40	75

^a^Lower Layer Super Output Area (LSOA): census reporting units containing between 1000 and 3000 individuals

^b^Population density: number of persons per hectare; used as a proxy measure of residential density

^c^Intersection density: the number of 3-way junctions standardised by LSOA area; used as a measure of street connectivity

^d^Index of Multiple Deprivation Score (IMD) [45]: an area deprivation score that combines several indicators of deprivation including income, employment, health and crime. Higher scores indicate more deprived areas

^e^Normalised Difference Vegetation Index (NDVI): a validated normalised scale of healthy vegetation cover; used as a measure for presence of greenery at the neighbourhood-level. Higher scores indicate areas with more healthy vegetation cover

^f^WalkScore uses a Google search algorithm to calculate a weighted score (1–100) based on the number and accessibility of amenities (such as shops and parks) within a 1-mile radius of a user-entered postcode, whereby closer amenities with the most accessible walking routes are weighted more strongly; used as a measure of ‘access to/ availability of destinations and services’. Higher scores indicate more ‘walkable’ areas

### Logic model

**Fig. 7:**
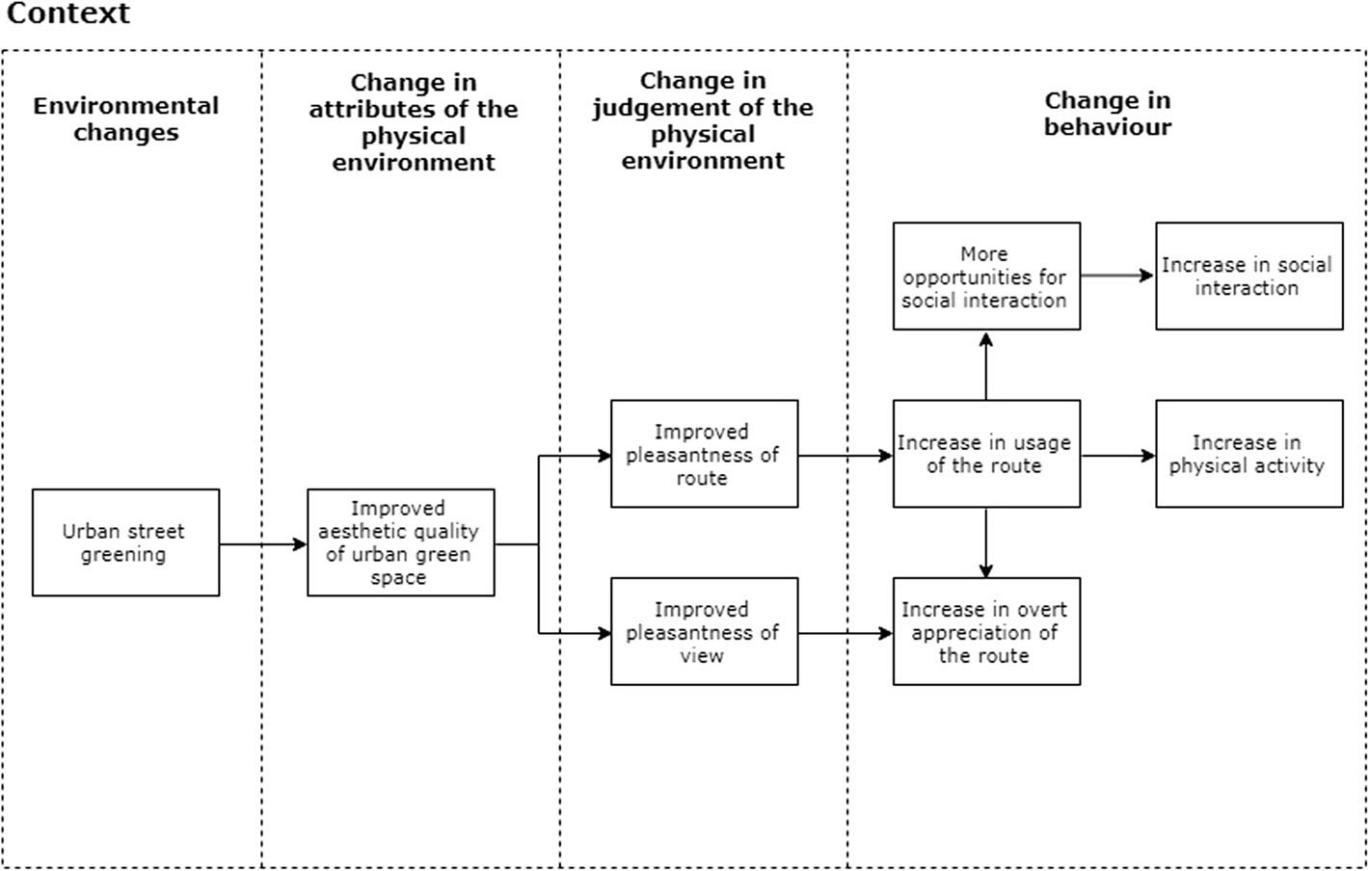
Urban street greening logic model

Hypothesised causal pathways are outlined in the logic model (Fig. 7), which is based on the framework suggested by Panter et al. [47]. It is proposed that improvements in the aesthetic quality of green space will increase overt appreciation in the intervention sites. The interventions are hypothesised to influence only one known variable associated with physical activity: aesthetic quality of the route. More aesthetically pleasing streetscapes, including the presence of attractive and well-maintained trees and greenery, are valued by older adults in facilitating physical activity [37]. The artificial tree decorations (e.g. tree socks) will be present all year round, ensuring the presence of the intervention in colder seasons.

#### Confounders

##### Weather

Observations will be carried out regardless of weather conditions, unless weather conditions become so extreme that they compromise the observer’s safety. To control for the confounding influence of weather, the observer will record the duration of any precipitation that occurs during each observation period. These data will inform a sensitivity analysis.

##### Other known confounders

Data collection at all time points will be carried out during UK school term dates to control for the change of activity around school holidays. Data collection is planned so that it does not overlap with the daylight saving clock change on 25th March 2018. A liaison from Southway Housing Trust will be contacted before data collection at each time point to enquire about any unrelated significant events that could influence the outcomes, such as other unrelated planned changes to the built environment. Media outlets (e.g. Twitter) will also be monitored to check for any significant events near to sites during data collection periods at each time point. Where possible, data collection will be arranged so that it does not co-occur with any unrelated significant events.

### Analysis plan

#### Inter-rater reliability

High inter-rater reliability for MOHAWk has previously been established between pairs of observers across three studies for assessing people’s behaviours and their characteristics (ICCs > 0.8) [33]. Inter-rater reliability will be calculated between each pair of observers at each time point to assess agreement on demographic and activity categories. Inter-rater reliability will be analysed using single rater two-way random effects ICC and percentage agreement.

#### Main analysis

The analysis will estimate the effect of the intervention on the number of older adults using the site at each of the two time points, and the types of behaviours they engage in per observation period, compared to the two corresponding comparison sites. The analysis will control for known confounders including weather, day and time. The primary analysis will analyse data for older adults and a secondary analysis will analyse data for all adults using the same methods.

The primary outcome will be a count per observation period of Take Notice behaviour at 12 months. Take Notice behaviour is the primary outcome because the interventions are expected to improve the aesthetic quality of green space by providing visual information of value to users, thus causing more overt appreciation in the intervention sites. The secondary outcome will be the overall count of older adults per observation period. Additional exploratory analyses will assess a count per observation period separately for each physical activity level (Sedentary, Walking, Vigorous) and Connect behaviour.

For each outcome we will follow three steps. Firstly, using a dataset that only includes the baseline data, and is blinded to group allocation, we will build a regression model to examine the relationship between the baseline count outcome and the covariates (weather, day, time). The overall count of older adults per observation period will be used as an additional covariate when analysing each of the behaviours (i.e. Sedentary, Walking, Vigorous, Take Notice, Connect). We will consider this count outcome as either a poisson distribution, a zero-inflated poisson or a normal distribution. We will consider all these approaches, and we will choose the most suitable approach by seeing which model has the best fit, assessed using Akaike’s information criterion (AIC) and measures of over-dispersion. Secondly, once we have developed a suitable model with the baseline data, we will combine the pre- and post-intervention data together and apply the model from step one. Thirdly, we will add into the model Group (intervention or comparison) and Period (pre or post). The treatment effect will be the coefficient for the interaction of Group and Period. This is a form of Difference in Differences analysis [48, 49], adapted because we do not have a before and after measure at the level of the hour.

#### Sensitivity analysis

A sensitivity analysis will be conducted to assess for any potential bias in the analysis of the primary and secondary outcome.

Observation periods will be removed for the sensitivity analysis if there is any precipitation that lasts for more than 50% of the observation period i.e. an overall accumulated duration of 30 min or more (recorded by the observer). This is in line with recommendations from MOHAWk [33].

Analysis will be undertaken using SPSS version 23 or later.

### Power calculation

Given the lack of previous studies of the causal effects of urban street greening, it is difficult to estimate the plausible size of the effect that interventions will have on the outcomes. However, the interventions only target one known variable that can influence physical activity and wellbeing within the context of a broader complex ‘system’ [50]; therefore, the effects on the measured outcomes, particularly physical activity, are likely to be small.

To assess the power of the study, we conducted a power calculation of the primary outcome measure: counts per observation period (hour) of Take Notice behaviour in older adults. We used the approach suggested by Donner and Klar ([51], p.66) for calculating the sample size for a matched pair design: calculate the number of clusters required for a completely randomised cluster design, and then multiply that by one minus the correlation between the mean outcomes in the two groups. This suits our context because it allows us to account for multiple comparison groups for each intervention group. We used the means and standard deviations (SD) from the two sites in the feasibility study. We assumed that one site was the ‘comparison’ group (i.e. the intervention site at baseline) and the other site was the ‘intervention’ group (this site had two benches, more greenery and was thus more aesthetically appealing based on ratings using the NGST). For the SDs for each site, we used the 80% upper one-sided confidence limit of the SD to account for the possibility that the SD from the feasibility data may be an underestimate.

A power calculation showed that if we match four intervention sites and eight comparison sites (12 in total) and have eight observation periods per cluster, we will have 99% power (*p* = .05, two-tailed test) to detect a difference between 0.5 (SD = .77) counts per observation period of Take Notice behaviour in older adults in the control group and 1.6 (SD = 1.72) in the intervention group. This assumes the ICC is 0.02, *p* = .05, two-tailed test: this low ICC value has been used in previous studies that have evaluated homogeneous parks (B. Han, personal communication). This also assumes that the ‘comparison’ group and ‘intervention’ groups from the feasibility study accurately represent the comparison and intervention sites in the main study at follow-up. However, due to the lack of data from previous studies on changes in older adult’s Take Notice behaviours in urban street greening interventions, data from the feasibility data was the most suitable available data to inform the power calculation.

## Discussion

This natural experimental study permits a rare and valuable opportunity to evaluate the causal effects of ‘real life’ changes in four small urban green spaces, within a deprived neighbourhood in an understudied population (older adults) and setting (UK). The findings will be useful for policy- and decision-makers in GM, as well as other urban areas in the UK and elsewhere in Europe.

This study will provide important methodological contributions by addressing seven out of eight key methodological weaknesses identified in a recent review to reduce bias in natural experimental studies [24] based on MRC guidance [20]. Bias due to confounding is a particularly pervasive problem in natural experimental studies [20]. We reduced the risk of bias due to confounding by developing a rigorous approach to comparison site matching and using appropriate statistical analyses to control for important known potential confounders (e.g. weather). Strengths of our novel approach to comparison site matching include the use of several objective and subjective variables at different levels of the environment (neighbourhood and street level) and multiple comparison sites to increase the likelihood of finding balanced comparison groups. Other methodological improvements in the present study include a published study protocol with a priori analyses specified, pioneering a newly developed tool to objectively measure physical activity and other wellbeing-related behaviours, clear reporting of interventions in line with the TIDieR checklist and a power calculation.

There is a lack of evidence and understanding on what specific kinds of changes in urban green space produce which outcomes in different contexts [47] i.e. what works, for whom and in what circumstances? [52]. The present study will address this issue by formally measuring the specific environmental change (i.e. aesthetic quality) using a UK validated tool for measuring the quality of green space (NGST). Using an objective outcome measure that is directly measured within the environmental context of interest will enable us to more confidently attribute changes in outcomes to the environmental change. This study will therefore provide an accurate insight into the effects of urban street greening on older adults’ physical activity and wellbeing in a deprived urban neighbourhood in the UK i.e. helping to answer what works, for whom and in what circumstances.

Understanding the pathways underlying the potential link between changes in urban green space (exposure) and physical activity and wellbeing (outcome) is important from a theoretical point of view, but also in terms of translating evidence into intervention or policy change [53]. However, there has been limited consideration and measurement of how changes in urban green space may work to change behaviour, particularly physical activity, in existing studies to date [15]. We developed a logic model explaining how the interventions are expected to influence older adults’ physical activity and wellbeing. We will seek to conduct a qualitative process analysis testing this logic model in a separate sub-study.

This study will use a parallel-groups design with a binary distinction between exposed intervention and unexposed comparison groups. A common difficulty when using this type of design is finding equally matched comparison sites. However, this design is suitable for the present study because intervention sites are located on residential streets; a type of land-use frequently found across the majority of neighbourhoods, thus providing ample unexposed potential matches. An alternative type of comparison site that can be used in natural experimental studies involves graded measures of exposure [54], such as distance from the intervention, as recommended by MRC guidance [20]. However, the interventions in this study are small; it is therefore unlikely that there will be any meaningful variation in exposure outside of the intervention site and thus graded measures of exposure would be less suitable.

## Conclusion

This study permits a rare opportunity to carry out a natural experimental study in the ‘real world’ as part of a multi-sectoral interdisciplinary collaboration. This study will also demonstrate the feasibility of incorporating rigorous methodology into the challenging field of natural experimental studies. As a result, this study will produce unique robust evidence on the causal effect of changes to urban green space in the UK on physical activity and wellbeing, in a growing but understudied ageing population in the context of environmental interventions.

## Additional files

Additional file 1: Comparison site matching process. (DOCX 59 kb)

Additional file 2: MOHAWk observation manual. (DOCX 1653 kb)

Additional file 3: Target area boundaries for each intervention and comparison site. (DOCX 10549 kb)

## Abbreviations

AIC: Akaike’s information criterion
ICC: Intraclass correlation coefficient
IMD: Index of multiple deprivation
LSOA: Lower layer super output area
MOHAWk: Method for Observing pHysical Activity and Wellbeing
MRC: Medical research council
NEF: New Economics Foundation
RCT: Randomised controlled trial
SD: Standard deviation
SOPARC: System for Observing Play and Recreation in Communities
UK: United Kingdom
US: United States
