# Supplementary material for: Evaluating the impact of improvements in urban green space on older adults’ physical activity and wellbeing: protocol for a natural experimental study
Source: BMC Public Health. 2018 Jul 27;18:923. doi: 10.1186/s12889-018-5812-z (PMC6062989; doi:10.1186/s12889-018-5812-z)
Supplement: Supplementary file 1 — Comparison site matching process. (DOCX 59 kb) [file 12889_2018_5812_MOESM1_ESM.docx]

**Comparison site matching process**

The overall aim of the comparison site matching process was to identify two closely matched comparison sites for each intervention site, using several key objective and subjective environmental variables.

***Step one: Matching at the neighbourhood (LSOA) level***

Each intervention site is located along a residential street, so the overall aim of the process was to identify the most closely matched streets based on key variables; comparison sites could then be identified within these streets. The first step was to identify the most closely matched neighbourhoods in which the potential streets for comparison sites could be searched from.

Due to a lack of available walkability indices for Greater Manchester (GM) it was necessary to manually search for neighbourhoods based on available spatial data at the Lower Layer Super Output Area (LSOA) level (census reporting units containing between 1000 and 3000 individuals) [1,2]. Population density, defined as the number of persons per hectare, was used as a proxy measure of residential density. Street connectivity was measured using street intersection density; the number of 3-way junctions standardised by LSOA area. Normalised Difference Vegetation Index (NDVI) scores, a normalised scale of healthy vegetation cover [3], were used for presence of greenery. Socioeconomic status was measured using the Index of Multiple Deprivation (IMD) Score [4]; an area deprivation score that combines several indicators of deprivation including income, employment, health and crime. Spatial analyses were carried out using ArcGIS 10.4.

To identify the most closely matched LSOAs to the intervention LSOA, a systematic funneling approach was used. All LSOAs in GM were firstly ranked in order of residential density and the 100 most closely matched LSOAs to each intervention site LSOA were extracted. These 100 LSOAs were then ranked in order of street connectivity and the 50 most closely matched LSOAs were selected. These two variables were matched first because they are the strongest and most consistent correlates of older adults’ physical activity [5,6]. Next, the remaining 50 LSOAs were ranked in order of closeness of IMD score to the intervention LSOA and the most closely matched 25 LSOAs were selected. Finally, these 25 LSOAs were ranked in order of those most closely matched on NDVI scores to the intervention LSOA and the five most closely matched LSOAs were selected. Thus, by the end of this step there were a manageable total of five potential LSOAs for each intervention site.

***Step two: Matching at the street level (access to/ availability of destinations and services)***

As there were no data available for ‘access to/ availability of destinations and services’ at the LSOA level, the second step was to measure this variable at the street level. There is a lack of data on walkability scores in GM and manually calculating distances to nearby destinations and services for each street within each LSOA was beyond the scope of this project. Instead, Walk Score (www.walkscore.com) was the most appropriate objective and reliable measure readily available. Walk Score is a website that uses a Google search algorithm to calculate a weighted score (1-100) based on the number and accessibility of amenities (such as shops and parks) within a 1-mile radius of a user-entered postcode, whereby closer amenities with the most accessible walking routes are weighted more strongly. Walk Scores have shown good correlation with gold-standard measures of walkability using Geographic Information Systems (GIS) [7and have previously been used in studies in the US [e.g. 8]. Walk Score confirmed that data is available in in the UK (WalkScore, personal communication).

To calculate Walk Scores for each street in each LSOA, it was necessary to obtain all postcodes within each LSOA. Postcodes were obtained using FreeMapTools (www.freemaptools.com): a free website that enables users to search for all postcodes within a user-defined area in the UK. Walk Scores were then calculated for each intervention site postcode and all postcodes extracted from the potential comparison site LSOAs. All postcodes were then ranked in order of closeness of Walk Score to each intervention site.

***Step three: Matching at the street level (streetscape characteristics)***

The aim of step three was to find at least three potential comparison sites located within streets (postcodes) most closely matched to the intervention site based on streetscape characteristics. All postcodes were remotely audited using Google Street View. Google Street View can be accessed via Google Maps ([www.maps.google.com](http://www.maps.google.com)) and permits users to remotely navigate 360° through panoramic images of the streetscape environment from the internet. Virtual streetscape audits were preferred given the vast number of postcodes that needed to be examined (between 165 and 212 postcodes for each intervention site). Empirical research has previously demonstrated that Google Street View is mostly a reliable and efficient tool to measure the streetscape in comparison with physical on-site audits [9].

Starting with postcodes most closely matched to the intervention site based on Walk Scores, JB audited the following objective streetscape characteristics associated with older adults’ physical activity for each postcode: road type (proxy measure of traffic), number of pedestrian access points (proxy measure of connectivity), presence of a pavement/ footpath, number of benches, presence of street lights, non-residential buildings, and presence of greenery. Presence of greenery, specifically a grass-covered area, was a required characteristic for potential comparison sites because all intervention sites contained a grass-covered area at baseline. For some of the intervention sites, it was necessary to iteratively search for more potential comparison sites; this was done by identifying more LSOAs and repeating the first three steps. By the end of step three, all intervention sites had three potential comparison sites.

***Step four: On-site environmental audits***

The aim of step four was to ensure potential comparison sites were closely matched in terms of quality and quantity of green space and pavements/ footpaths, and attractiveness of the buildings. These variables are more difficult to reliably judge using Google Street View and therefore required on-site audits of the environment.

JB visited each potential comparison site to systematically audit each site using two validated environmental audit tools: the Neighbourhood Green Space Tool (NGST) [10] and the 54-item abbreviated version of the Microscale Audit of Pedestrian Streetscapes (MAPS-Abbreviated) measure of street design [11]. NGST enabled an audit of the green space characteristics and was specifically developed in the UK; which was important due to the lack of environmental walkability tools that have been developed in the UK [12]. MAPS-Abbreviated was chosen because it measures the pedestrian streetscape (including pavements/ footpaths and buildings) and was partly based on a previous tool that has been modified by the Healthy Aging Network [13]. The scores on both tools in each potential comparison site and corresponding intervention site were used to inform selection of the two final comparison sites.

***Step five: Matching on pedestrian traffic***

The fifth and final step aimed to ensure that intervention and comparison sites were well matched in terms of pedestrian traffic; that is, the frequency of users passing through a site. It has been found that 15 minutes of observation can provide excellent reliability in estimating the frequency of users passing through a site across the whole hour [Benton et al., in press]. Therefore, 15 minute observations were carried out across the 12 sites (four intervention and eight comparison sites) during August 2017 to count pedestrian traffic. JB conducted 15 minute observations at two intervention sites on a Thursday between 12-2pm, and did the same at the other two intervention sites on a Friday between 12-2pm. Observations of the corresponding comparison sites were then carried out in the following week on Thursday and Friday between 12-2pm. All final comparison sites were, for the most part, similar to corresponding intervention sites in terms of pedestrian traffic and subjective ‘feel’ of the sites.

**References**

1. Office for National Statistics. 2001 Census: Digitised Boundary Data (England and Wales) [computer file]. UK Data Service Census Support. Downloaded from: http://edina.ac.uk/census. Licensed under the terms of the Open Government Licence [http://www.nationalarchives.gov.uk/doc/open-government-licence/ version/2]
2. Office for National Statistics. Super Output Areas: Office for National Statistics, 2011
3. Markevych I, Schoierer J, Hartig T, Chudnovsky A, Hystad P, Dzhambov AM, De Vries S, Triguero-Mas M, Brauer M, Nieuwenhuijsen MJ, Lupp G. Exploring pathways linking greenspace to health: Theoretical and methodological guidance. Environ Res. 2017;158:301-17.
4. Department for Communities and Local Government. 2015. English Indices of Deprivation 2015 [computer file]. Downloaded from: https://www.gov.uk/government/statistics/english-indices-of-deprivation-2015. Licensed under: https://www.nationalarchives.gov.uk/doc/open-government-licence/version/3
5. Barnett DW, Barnett A, Nathan A, Van Cauwenberg J, Cerin E. Built environmental correlates of older adults’ total physical activity and walking: a systematic review and meta-analysis. Int J Behav Nutr Phys Act. 2017;14(1):103.
6. Cerin E, Nathan A, Van Cauwenberg J, Barnett DW, Barnett A. The neighbourhood physical environment and active travel in older adults: a systematic review and meta-analysis. Int J Behav Nutr Phys Act. 2017;14(1):15.
7. Carr LJ, Dunsiger SI, Marcus BH. Validation of Walk Score for estimating access to walkable amenities. Br J Sports Med. 2011;45(14):1144-8.
8. Althoff T, Sosič R, Hicks JL, King AC, Delp SL, Leskovec J. Large-scale physical activity data reveal worldwide activity inequality. Nature. 2017;547(7663):336-9.
9. Badland HM, Opit S, Witten K, Kearns RA, Mavoa S. Can virtual streetscape audits reliably replace physical streetscape audits?. J Urban Health. 2010;87(6):1007-16.
10. Gidlow CJ, Ellis NJ, Bostock S. Development of the neighbourhood green space tool (NGST). Landsc and Urban Planning. 2012;106(4):347-58.
11. Cain KL, Gavand KA, Conway TL, Geremia CM, Millstein RA, Frank LD, Saelens BE, Adams MA, Glanz K, King AC, Sallis JF. Developing and validating an abbreviated version of the Microscale Audit for Pedestrian Streetscapes (MAPS-Abbreviated). J Transport & Health. 2017; 5: 84-96.
12. Brownson RC, Hoehner CM, Day K, Forsyth A, Sallis JF. Measuring the built environment for physical activity: state of the science. Am J Prev Med. 2009;36(4):S99-123.
13. Kealey M, Kruger J, Hunter R, Ivey S, Satariano W, Bayles C, Ramirez B, Bryant L, Johnson C, Lee C, Levinger D. Engaging older adults to be more active where they live: audit tool development. Prev Chronic Dis. 2005; 2:1–2.
