# Supplementary material for: Evaluating the impact of improvements in urban green space on older adults’ physical activity and wellbeing: protocol for a natural experimental study
Source: BMC Public Health. 2018 Jul 27;18:923. doi: 10.1186/s12889-018-5812-z (PMC6062989; doi:10.1186/s12889-018-5812-z)
Supplement: Supplementary file 2 — MOHAWk observation manual. (DOCX 1653 kb) [file 12889_2018_5812_MOESM2_ESM.docx]

**MOHAWk**

**(Method for Observing Physical Activity and Wellbeing)**

**Observation Manual**

**(Preliminary version for the purpose of the protocol for the GHIA natural experimental study^[[1]](#footnote-1)^)**

Revised 19/01/2018

**CONTENTS**

**Page**

1. [**OVERVIEW OF MOHAWk 3**](#Overview_of_MOHAWk)
2. [**OBSERVATION PREPARATION 4**](#Observation_preparation)
3. [**CODES AND RECORDING 5**](#Codes_and_recording)
4. [**OBSERVATION PROCEDURES 10**](#Observation_procedures)
5. [**OBSERVATION PERIODS 12**](#Observation_periods)
6. [**DATA ANALYSIS 13**](#Data_analysis)
7. [**COMPARISON WITH EXISTING OBSERVATION TOOLS 13**](#Comparison_with_existing_observation_too)

[**APPENDIX 1 – MOHAWk observation form 15**](#Appendix_1)

[**APPENDIX 2 – MOHAWk data summary form 21**](#Appendix_2)

[**APPENDIX 3 – Example of a completed MOHAWK observation form 22**](#Appendix_3)

[**APPENDIX 4 – Example of a completed MOHAWK data summary form 23**](#Appendix_4)

1. **OVERVIEW OF MOHAWk**

**Introduction to MOHAWk**

Method for Observing Physical Activity and Wellbeing (MOHAWk) is an observation tool designed for measuring physical activity and wellbeing in small urban green space such as pocket parks, tree-lined streets, and green corridors along waterways. Using direct, systematic observation of behaviour, it provides an assessment of the number of people moving through a predetermined area (Target Area), their characteristics and activities they engage in. Using the tool, trained observers measure people’s physical activity levels (three intensities: Sedentary, Walking and Vigorous), two behavioural indicators of wellbeing (Connect: connecting with others; and Take Notice: taking notice of the environment), activity type, gender, age, ethnicity and whether they are overweight or have a disability (require assistance to move). Observers also document weather conditions (duration of any precipitation) and any incivilities in the Target Area.

**Summary of how it works**

MOHAWk applies interval time sampling techniques using continuous scanning of activities and characteristics of all individuals entering pre-determined boundaries within public open space (Target Area). Observers record activity in the Target Area during hour-long observation periods using a standardised observation form (see Appendix 1).

Each observed individual is coded for the following information:

- Physical activity level (Sedentary, Walking, Vigorous)
- Behavioural indicators of wellbeing (Connect, Take Notice)
- Activity type (Cycling, Using Phone, Headphones, Dog Walking or other activities)
- Gender (Male, Female)
- Ethnicity (White, Non-White)
- Age (Infant, Child, Teen, Adult, Older Adult)
- Overweight (Yes/ No)
- Disablility (Yes/ No)

The following contextual information is also recorded:

- Extent of the following incivilities in the Target Area:
  - General litter
  - Evidence of alcohol use (empty bottles/ cans)
  - Evidence of drug taking (e.g., needles, syringes)
  - Graffiti
  - Broken glass
  - Vandalism
  - Dog mess
  - Noise
- Weather (duration of any precipitation during the observation period)

1. **OBSERVATION PREPARATION**

***Defining the Target Area***

Before data collection begins, the site should be visited by all observers, who should agree on the precise boundaries of the Target Area, so that it is possible for anyone to agree on whether an individual falls within the boundary of that area or not. Any individual and their behaviour should only be coded within this Target Area.

A Target Area should be large enough to accommodate activity, but small enough so that observers can accurately count everyone who passes through the Target Area. The Target Area should be located where activity is most likely to occur, whilst considering an appropriate size of Target Area where observers can reliably view and code the characteristics and activity of all individuals in that area. This tool is most suitable for small urban spaces, so multiple Target Areas are not appropriate.

A location for the researcher to carry out observations should be identified, with a good view of the Target Area and no visual obstructions. Ideally, this should include an area with good cover for adverse weather. If an area that affords a good view with good cover is not possible, an area that affords a reasonable view with good cover should be identified for particularly severe weather. This is important because observations are carried out regardless of weather conditions, unless weather conditions become so extreme that they compromise the observer’s safety.

Existing boundaries (e.g., tree lines, fences, road junctions) can help define a Target Area. However, these boundaries need to be the same throughout all observation periods.

If dangerous areas are to be observed, do this from a safe distance. At least one “safe” area should be identified, where the researcher can go if anyone being observed becomes aggressive e.g., café, public library, busy public space.

***Before observing the Target Area***

All observers should be fully trained in using MOHAWk. Ideally, all observers should be trained together and should carry out practice observations prior to the ‘real’ observations. This will help calibrate observers and enable observers to agree on any ambiguities; thus improving inter-rater reliability. Inter-rater reliability calculations should be conducted to formally assess whether observers are achieving acceptable agreement with each other e.g., using intraclass correlation coefficients (ICC).

Print out a copy of the site map so that the Target Area can be clearly defined. Also, print several copies of a letter detailing information about the study (including details of ethical approval), in the event that any members of the public request more information about what observers are doing.

It is recommended that photographs and environmental audits of the Target Area are taken prior to observations. An environmental audit of the Target Area should be carried out using a validated tool appropriate for the particular area under investigation and the purposes of the study.

Observers should arrive at the site at least 15 minutes prior to the official start of coding. In this time before observations begin, observers should record the extent of incivilities for each Target Area (General litter, Evidence of alcohol use, Evidence of drug taking, Graffiti, Broken glass, Vandalism, Dog mess, Noise – see next section).

1. **CODES AND RECORDING**

***Target Area incivilities***

Tick “None”, “Hardly noticeable”, “Noticeable”, or “Very Noticeable” to describe specific conditions for each Target Area.

Scoring for the extent of incivilities should be based on whether people using the green space would be generally aware of its presence (observers should not have to forensically examine all parts of the green space).

| **Incivility** | **None** | **Hardly noticeable** | **Noticeable** | **Very noticeable** |
| --- | --- | --- | --- | --- |
| **General litter** | None visible | Hardly noticeable  A few items visible on  the ground | Noticeable  Several items are on  the ground | Very noticeable Many  items are on the  ground |
| **Evidence of alcohol**  **use** (bottles, cans, or bottle caps visible) | None visible | Hardly noticeable  A few items | Noticeable  Several items | Very noticeable |
| **Evidence of drug**  **taking**  (e.g., paint cans, rags, baggies, rolling papers) | None visible | None visible | One or more clear  examples of drug  taking | Very noticeable |
| **Graffiti** | None visible | Hardly noticeable, but it appears on up to a few pieces of furniture/ equipment | Noticeable  Several small or large pieces, clearly visible | Very noticeable Several large pieces, on much of the park furniture/ equipment |
| **Broken glass** | None visible | A few pieces of broken glass, does not really spoil enjoyment of space | Several pieces of broken glass, affecting enjoyment of area | Many pieces of broken glass, affecting enjoyment of area |
| **Vandalism** | None visible | Hardly noticeable, but some evidence on up to a few pieces of furniture/ equipment | Noticeable, more than a few pieces of equipment **OR** an area of the space has been rendered unusable by vandalism | Very noticeable, more equipment in disrepair than in good order because of vandalism. Signs of vandalism are obvious |
| **Dog mess** | None visible | Hardly noticeable, perhaps a single example | Noticeable/ several dog refuse piles, affecting enjoyment of area | Very noticeable,  seriously affecting  enjoyment of area |
| **Noise** | Not aware of any | Some sound but hardly noticeable, not annoying | Sound(s) is (are) noticeable and interfere(s) with enjoyment of area | Noticeable sounds which are unpleasant. Seriously affecting enjoyment of area |

*Taken from: Gidlow CJ, Ellis NJ, Bostock S. Development of the neighbourhood green space tool (NGST). Landscape and Urban Planning. 2012;106(4):347-58.*

***Gender***

Individuals are coded as either Male or Female.

***Age Group***

Individuals are coded by age group according to the following criteria:

Infant = Babies or toddlers for whom it is difficult to judge gender or ethnicity.

NOTE: Clues indicative of infants include children being carried or in a pram, unless their gender and ethnicity is clear.

NOTE: Do not code gender, ethnicity or activity level for infants.

Child = Individuals from infancy to 12 years of age.

NOTE: Children can be identified by general appearance, especially if they are wearing a school uniform. Children are more likely to be accompanied by a parent.

Teen = Individuals aged 13 to 20 years of age

NOTE: Teens are likely to dress more ‘extravagant’ than children under 12 years of age. Any individual that appears like a secondary school student, college student or university undergraduate can be coded as a Teen.

Adult = Individuals aged 21 to 59 years of age.

Older Adult = Individuals aged 60 years of age and older.

NOTE: Individuals coded as an Older Adult will be those who appear to be 60 years or older based on their general appearance and mobility. The main criteria observers should take note of is gait and general movement, as Older Adults may show signs of aging with stiffer, slower, or simply inhibited movements. Observers should also look for physical attributes traditionally associated with age, such as grey hair, wrinkles, or lack of hair, to decide if issues with gait or mobility appear to be linked to advancing age or other causes of impairment. People who are wearing work-related uniforms should probably not be coded as an Older Adult.

***Ethnicity***

Code whether the ethnicity for each individual is White or Non-White. Whilst there may be ambiguities in deciding whether some ethnicities are White or Non-White (e.g., mixed ethnicity), such ambiguities should be agreed before observations begin and observers should be consistent in coding ethnicity.

***Physical Activity Level***

Determine the activity level based on the following criteria:

**Sedentary (S) =** Individuals are lying down, sitting, or standing in place.

NOTE: Observers should consider whether that behaviour would be coded as Sedentary if they conducted one brief scan of the Target Area; if the answer is yes, then it is likely that the behaviour can be coded as Sedentary. Very brief or incidental instances of sitting or standing should not be coded as Sedentary behaviour. For example, the following examples usually would not be coded as Sedentary as these are brief or incidental behaviours: briefly stopping to pick up a piece of litter, briefly waiting for a car to pass before crossing the road. Whereas the following examples would usually be coded as Sedentary as these are prolonged or deliberative behaviours: stopping to talk to someone, waiting at a pedestrian crossing for several seconds.

NOTE: If an individual in a pram is coded as a Child, then they should be coded as Sedentary. The activity level for infants is not coded.

**Walking (W)** = Individuals are walking at a casual pace.

**Vigorous (V)** = Individuals are currently engaged in an activity more vigorous than an ordinary walk (e.g., increasing heart rate causing them to sweat, such as jogging, power walking, doing cart wheels, skipping). All cyclists should be coded as Vigorous, unless they are walking with their bicycle in which case they should be coded as Walking.

NOTE: When a person cycles into the observation area, dismounts, locks cycle and then walks away, then it may be appropriate for all three physical activity behaviours to be coded i.e., Sedentary, Walking and Vigorous.

NOTE: Individuals in a mobility scooter or electric wheelchair should be coded as Sedentary, whereas those in a manual wheelchair should be coded as Walking or Vigorous depending on how fast they are moving.

***Behavioural indicators of wellbeing***

**Take Notice (TN)** = Individuals appear as if they are making a conscious decision to appreciate their surroundings. Examples of this could be (a) extended viewing of the scenery, (b) an intentional pause in activity to look at or photograph something in the vicinity, or (c) a pronounced head swivel to look at a specific object, view or person.

The following scenarios are not to be coded as Take Notice behaviours:

- Staring into space (e.g. when smoking or looking closely at phones).
- Looking around when crossing a road, junction, pathway or other crossing.
- An individual is on a phone call.
- Taking notice of the researcher.

NOTE: If there is uncertainty whether an individual engaged in Take Notice behaviour, then it is unlikely that this is sufficient to be coded as Take Notice.

**Connect (C)** = Individuals are engaging/ interacting with a person or the people around them in some way. The activity must involve either (a) conversing (e.g., talking and listening, or using sign language) with other users, (b) being physically linked with someone (e.g., holding hands, linked arms, being carried on shoulders), (c) smiling and making eye contact when passing through a door or other narrow space, or (d) participation in a group activity. Note that when two people are interacting, this should be coded as two behaviours i.e., one for each individual person.

The following scenarios are not to be coded as Connect behaviours:

- Individuals who are not physically or verbally interacting with others in the same vicinity (e.g., phone call, video call).
- Walking or cycling side by side.
- Interacting with the researcher.

NOTE: Similar to some Sedentary behaviours, Take Notice and Connect behaviours are sometimes momentary. Therefore, observers should consider whether that behaviour would be coded as Take Notice or Connect if they conducted one brief scan of the Target Area; if the answer is yes, then it is likely that the behaviour can be coded as Take Notice or Connect.

***Activity Type***

There are four predefined types of activity that observers can record during observations:

- Cycling (riding, walking or standing with a bicycle)
- Using Phone (holding and using a phone in some way e.g., texting, phone call, taking a picture or recording a video)
- Headphones (wearing headphones)
- Dog Walking (walking with a dog, whether it be on or off a lead)

NOTE: multiple people walking the same dog can all be coded as Dog Walking e.g., if two individuals are walking one dog then both individuals should be coded as Dog Walking

There is space on the MOHAWk data collection form to add further activities that researchers may be interested in coding. For instance, researchers may be interested in activities specific to a particular site e.g., fishing may be a prominent activity of interest when observing along a canal waterway (see Appendices 3 and 4 for an example). Alternatively, researchers might want to code specific Connect (e.g., holding hands) or Take Notice (e.g., taking photographs) behaviours.

***Group/ Busy***

*Large groups*

When there is a large group of people (~>10), observers should only record the number of people in that group and record estimates of the frequency (or percentage if the overall count is known) of each activity level and category for age, gender, ethnicity, overweight and disability (see Appendices 3 and 4 for an example).

When using this procedure for large groups, observers should prioritise estimates for the most important variables depending on the primary focus of the evaluation. For example, if the primary aim of the evaluation is to evaluate the impact of an intervention specifically on women’s’ physical activity levels, then prioritise estimates of physical activity levels and gender.

*Busy periods*

The procedure for dealing with large groups can also be used during extremely busy periods as a last resort i.e., when it is impossible to reliably code the characteristics and activity of each separate individual. For example, if it is extremely busy between 3pm and 3:15pm, then observers should record the overall number of people

***Overweight***

Record whether the individual is overweight; defined as a weight that is perceived to impair individual’s health based on their height and type of body mass (large muscle mass is not classed as overweight). Examples of indicators of overweight individuals include stomach protruding over waistband and visible breathlessness when walking that is likely due to being overweight. Pregnant women are not classed as overweight. Given the arbitrary nature of judging an individual’s weight, observers should discuss examples to help calibrate and standardise coding for weight.

***Disability***

Record whether the individual has a physical disability i.e. requires assistance to move. For instance, use of a walking stick, wheelchair or mobility scooter. Individuals who are limping, but without a mobility aid, should not be classed as having a disability.

***Weather***

Record the duration (approximate start and end time) of any precipitation that occurs during the observation period.

***Comments***

Make a note of any other key observations that occur during the observation period that are relevant to the data e.g., if anyone spoke to the observer during the observation period, if any activities were particularly popular during the observation period, reasons for any missing data, toilet breaks.

1. **OBSERVATION PROCEDURES**

***How to observe***

Observers should continuously scan the Target Area for the full hour. When an individual enters the Target Area, observers should record their gender (Female, Male), age (Infant, Child, Teen, Adult, Older Adult), ethnicity (White, Non-White), activity type, activity level (Sedentary, Walking, Vigorous, Take Notice, Connect), and if they are overweight or have a disability (require assistance to move).

All individuals entering the Target Area are coded unless they are in a vehicle.

If an observed person reappears in the Target Area in the same observation period, do not record a second time.

The unit of coding is the behaviour, so that the number of people performing each behaviour should be counted. Therefore, the same person can be coded as engaging in multiple behaviours, but each behaviour cannot be coded more than once for the same person within the same observation period e.g., if a person speaks to someone and then hugs someone else within the same observation period, this would only be coded as one Connect behaviour for that person.

Observers have the option to make a mark on the observation form every 15 minutes during the hour-long observation period (see Appendix 3 for an example). This provides researchers with the option to analyse data by 15-minute blocks as well as hour-long observation periods.

If necessary, observers can move around the Target Area, as long as observers do not interfere with activity and only code individuals entering the Target Area.

It may be more difficult for observers to reliably code individual’s characteristics and their activity levels during adverse weather conditions e.g., people are more likely to wear protective clothing, such as hooded jackets, when it is cold or raining. However, observer should record their best estimate of individual’s characteristics and their activity levels to avoid missing data.

***Observation procedure***

1. Prior to the start of the observation period, on the observation form, record the Date/ Day, Site name, Observer initials, Start time and End time.
2. Score the extent of incivilities in each area (General litter, Evidence of alcohol use, Evidence of drug taking, Graffiti, Broken glass, Vandalism, Dog mess, Noise).
3. Record the gender, age, ethnicity, activity type and activity level of all individuals that enters the Target Area, and record if they are overweight or have a disability onto the MOHAWk observation form (see Appendix 1 for the form and Appendix 3 for an example).
4. After the observation period has finished, record total counts of gender, age, ethnicity, activity type, activity level, overweight and disability onto the MOHAWk summary form (see Appendix 2 for the form and Appendix 4 for an example).

***Toilet breaks***

A designated place for toilet breaks should be determined during the observation preparation stage. Ideally toilet breaks should be planned to occur before or after observation periods. However, should a toilet break be necessary then this should ideally be taken in the final minutes of the observation period. Any toilet breaks should be documented on the comments section of the MOHAWk data collection form.

***Respecting the public***

Whilst observers should avoid standing out, observers should be overt about what they are doing (e.g. wear a high visibility vest, use a clipboard) and should be willing to respond honestly to any questions from members of the public during observations. Observers should ensure they respect members of the public and politely engage with them, even if it means that they miss individuals entering the Target Area. If any members of the public (particularly local residents) request that the observer should stop, then the observer should immediately stop and postpone all planned future observations at that particular site.

1. **OBSERVATION PERIODS**

***Timing and frequency of Observation Periods***

Observation periods are one hour long. Observation periods can be carried out at any time of the day depending on the aims and requirements of the evaluation, and available time and resources. For example, MOHAWk is being used for the Green Infrastructure and the Health and Wellbeing Influences on an Ageing Population (GHIA) study: a study evaluating the impact of improvements in urban green space on older adults’ physical activity and wellbeing. Observations for this study will be conducted at four set observation periods per day: morning (10-11am), lunchtime (12-1pm), afternoon (3-4pm), and evening (5-6pm). These times were found to capture the biggest variation in older adults’ activity across the day in a feasibility study, whilst also providing sufficient time for breaks and possible travel to other sites in between observation periods.

***Missing or Postponed Observation Periods***

Any missed observations (e.g., due to illness) should be rescheduled for the same day of the next available week e.g., if a 3-4pm observation period is missed on Monday, it should be made up on the next available Monday at 3-4pm.

***Example Observation Period***

9:45am - Check Target Area, prepare data forms and rate the extent of any incivilities in the Target Area

10am - Start observing in Target Area

11am - Stop observing in Target Area

11:01am - Transfer data from the MOHAWk observation form onto the MOHAWk summary form

1. **DATA ANALYSIS**

***Summarising data***

Researchers should use the MOHAWk summary form to summarise overall counts of gender, age, ethnicity, activity type, activity level, overweight and disability.

The MOHAWk summary form currently links age with gender, ethnicity, activity level, weight and disability. However, researchers can amend this form depending on their needs e.g., researchers can edit the form to link gender and activity level, for instance, if they specifically want to know how many males and females engage in vigorous activity.

***Sensitivity analysis***

MOHAWk methodology requires observers to carry on observing regardless of weather conditions, unless weather conditions become so extreme that they compromise the observer’s safety. Therefore, to control for the potential bias associated with weather, it is recommended that a sensitivity analysis is carried out to assess the impact of weather (specifically precipitation).

It is recommended that observation periods should be removed for the sensitivity analysis if there is any precipitation that lasts for more than 50% of the observation period i.e. an overall accumulated duration of 30 minutes or more (recorded by the observer).

1. **COMPARISON WITH EXISTING OBSERVATION TOOLS**

MOHAWk is based on similar principles as SOPARC (System for Observing Play and Recreation in Communities)^[[2]](#footnote-2)^: an existing observation tool for measuring physical activity in parks and communities. Similarities between MOHAWk and SOPARC include:

- The use of observations to obtain direct information on people’s characteristics and their physical activity behaviour;
- Hour-long observation periods;
- Same criteria for coding physical activity levels, age and gender.

However, there are numerous important differences between MOHAWk and SOPARC:

- MOHAWk observations are continuous, whereas SOPARC observations are based on momentary time sampling;
- MOHAWk also measures two behavioural indicators of wellbeing (Connect, Take Notice), and whether each individual is overweight or has a disability;
- SOPARC splits the area into multiple Target Areas, whereas MOHAWk is more suited towards smaller public spaces and thus does not require multiple Target Areas;
- SOPARC assesses age and ethnicity in separate scans to physical activity levels, whereas MOHAWk codes all activities and characteristics of each person simultaneously;
- MOHAWk permits observers to observe in whichever way suitable to the Target Area, whereas SOPARC specifies that observers scan from left to right;
- MOHAWk codes ethnicity as White or Non-White whereas SOPARC codes ethnicity as Latino, Black, White or Other;
- SOPARC specifies that observations are cancelled during adverse weather conditions, whereas MOHAWk observations are carried out during adverse weather conditions and a sensitivity analysis is recommended to control for the potential bias associated with weather;
- The recorded characteristics of the Target Area are different in MOHAWk and SOPARC.

Many of these differences are because MOHAWk was developed in the UK for small public spaces, whereas SOPARC was developed in the United States primarily for parks.

**APPENDIX 1 – MOHAWk observation form**

**START TIME:** **__________­­_______ END TIME:** **_________________**

**DATE / DAY: _____________________________________________ SITE: _______________________________________________ OBSERVER: ______________**

**WEATHER / COMMENTS: __**__**______________________________________________________________________________________________________________**

*Note any key observations. For weather, include the duration of any precipitation e.g., ‘Rain from 10.20-10.45am’*

| **Person** | **Gender** | | **Age Group** | | | | | **Ethnicity** | | **Activity Type(s)** | | | | | **Activity Level(s)** | | | | | **Group (record head count in group)** | **Over-weight?** | **Disability?** |
| --- | --- | --- | --- | --- | --- | --- | --- | --- | --- | --- | --- | --- | --- | --- | --- | --- | --- | --- | --- | --- | --- | --- |
|  | **Female** | **Male** | **Infant** | **Child** | **Teen** | **Adult** | **Older Adult** | **White** | **Non-White** | **Cycling** | **Using phone** | **Head-phones** | **Dog walking** | **……….** | **S** | **W** | **V** | **TN** | **C** |  |  |  |
| 1 |  |  |  |  |  |  |  |  |  |  |  |  |  |  |  |  |  |  |  |  |  |  |
| 2 |  |  |  |  |  |  |  |  |  |  |  |  |  |  |  |  |  |  |  |  |  |  |
| 3 |  |  |  |  |  |  |  |  |  |  |  |  |  |  |  |  |  |  |  |  |  |  |
| 4 |  |  |  |  |  |  |  |  |  |  |  |  |  |  |  |  |  |  |  |  |  |  |
| 5 |  |  |  |  |  |  |  |  |  |  |  |  |  |  |  |  |  |  |  |  |  |  |
| 6 |  |  |  |  |  |  |  |  |  |  |  |  |  |  |  |  |  |  |  |  |  |  |
| 7 |  |  |  |  |  |  |  |  |  |  |  |  |  |  |  |  |  |  |  |  |  |  |
| 8 |  |  |  |  |  |  |  |  |  |  |  |  |  |  |  |  |  |  |  |  |  |  |
| 9 |  |  |  |  |  |  |  |  |  |  |  |  |  |  |  |  |  |  |  |  |  |  |
| 10 |  |  |  |  |  |  |  |  |  |  |  |  |  |  |  |  |  |  |  |  |  |  |
| 11 |  |  |  |  |  |  |  |  |  |  |  |  |  |  |  |  |  |  |  |  |  |  |
| 12 |  |  |  |  |  |  |  |  |  |  |  |  |  |  |  |  |  |  |  |  |  |  |
| 13 |  |  |  |  |  |  |  |  |  |  |  |  |  |  |  |  |  |  |  |  |  |  |
| 14 |  |  |  |  |  |  |  |  |  |  |  |  |  |  |  |  |  |  |  |  |  |  |
| 15 |  |  |  |  |  |  |  |  |  |  |  |  |  |  |  |  |  |  |  |  |  |  |
| 16 |  |  |  |  |  |  |  |  |  |  |  |  |  |  |  |  |  |  |  |  |  |  |
| 17 |  |  |  |  |  |  |  |  |  |  |  |  |  |  |  |  |  |  |  |  |  |  |
| 18 |  |  |  |  |  |  |  |  |  |  |  |  |  |  |  |  |  |  |  |  |  |  |
| 19 |  |  |  |  |  |  |  |  |  |  |  |  |  |  |  |  |  |  |  |  |  |  |
| 20 |  |  |  |  |  |  |  |  |  |  |  |  |  |  |  |  |  |  |  |  |  |  |
| 21 |  |  |  |  |  |  |  |  |  |  |  |  |  |  |  |  |  |  |  |  |  |  |
| 22 |  |  |  |  |  |  |  |  |  |  |  |  |  |  |  |  |  |  |  |  |  |  |
| 23 |  |  |  |  |  |  |  |  |  |  |  |  |  |  |  |  |  |  |  |  |  |  |
| 24 |  |  |  |  |  |  |  |  |  |  |  |  |  |  |  |  |  |  |  |  |  |  |
| 25 |  |  |  |  |  |  |  |  |  |  |  |  |  |  |  |  |  |  |  |  |  |  |
| 26 |  |  |  |  |  |  |  |  |  |  |  |  |  |  |  |  |  |  |  |  |  |  |
| 27 |  |  |  |  |  |  |  |  |  |  |  |  |  |  |  |  |  |  |  |  |  |  |
| 28 |  |  |  |  |  |  |  |  |  |  |  |  |  |  |  |  |  |  |  |  |  |  |
| 29 |  |  |  |  |  |  |  |  |  |  |  |  |  |  |  |  |  |  |  |  |  |  |
| 30 |  |  |  |  |  |  |  |  |  |  |  |  |  |  |  |  |  |  |  |  |  |  |
| 31 |  |  |  |  |  |  |  |  |  |  |  |  |  |  |  |  |  |  |  |  |  |  |
| 32 |  |  |  |  |  |  |  |  |  |  |  |  |  |  |  |  |  |  |  |  |  |  |
| 33 |  |  |  |  |  |  |  |  |  |  |  |  |  |  |  |  |  |  |  |  |  |  |
| 34 |  |  |  |  |  |  |  |  |  |  |  |  |  |  |  |  |  |  |  |  |  |  |
| 35 |  |  |  |  |  |  |  |  |  |  |  |  |  |  |  |  |  |  |  |  |  |  |
| 36 |  |  |  |  |  |  |  |  |  |  |  |  |  |  |  |  |  |  |  |  |  |  |
| 37 |  |  |  |  |  |  |  |  |  |  |  |  |  |  |  |  |  |  |  |  |  |  |
| 38 |  |  |  |  |  |  |  |  |  |  |  |  |  |  |  |  |  |  |  |  |  |  |
| 39 |  |  |  |  |  |  |  |  |  |  |  |  |  |  |  |  |  |  |  |  |  |  |
| 40 |  |  |  |  |  |  |  |  |  |  |  |  |  |  |  |  |  |  |  |  |  |  |
| 41 |  |  |  |  |  |  |  |  |  |  |  |  |  |  |  |  |  |  |  |  |  |  |
| 42 |  |  |  |  |  |  |  |  |  |  |  |  |  |  |  |  |  |  |  |  |  |  |
| 43 |  |  |  |  |  |  |  |  |  |  |  |  |  |  |  |  |  |  |  |  |  |  |
| 44 |  |  |  |  |  |  |  |  |  |  |  |  |  |  |  |  |  |  |  |  |  |  |
| 45 |  |  |  |  |  |  |  |  |  |  |  |  |  |  |  |  |  |  |  |  |  |  |
| 46 |  |  |  |  |  |  |  |  |  |  |  |  |  |  |  |  |  |  |  |  |  |  |
| 47 |  |  |  |  |  |  |  |  |  |  |  |  |  |  |  |  |  |  |  |  |  |  |
| 48 |  |  |  |  |  |  |  |  |  |  |  |  |  |  |  |  |  |  |  |  |  |  |
| 49 |  |  |  |  |  |  |  |  |  |  |  |  |  |  |  |  |  |  |  |  |  |  |
| 50 |  |  |  |  |  |  |  |  |  |  |  |  |  |  |  |  |  |  |  |  |  |  |
| 51 |  |  |  |  |  |  |  |  |  |  |  |  |  |  |  |  |  |  |  |  |  |  |
| 52 |  |  |  |  |  |  |  |  |  |  |  |  |  |  |  |  |  |  |  |  |  |  |
| 53 |  |  |  |  |  |  |  |  |  |  |  |  |  |  |  |  |  |  |  |  |  |  |
| 54 |  |  |  |  |  |  |  |  |  |  |  |  |  |  |  |  |  |  |  |  |  |  |
| 55 |  |  |  |  |  |  |  |  |  |  |  |  |  |  |  |  |  |  |  |  |  |  |
| 56 |  |  |  |  |  |  |  |  |  |  |  |  |  |  |  |  |  |  |  |  |  |  |
| 57 |  |  |  |  |  |  |  |  |  |  |  |  |  |  |  |  |  |  |  |  |  |  |
| 58 |  |  |  |  |  |  |  |  |  |  |  |  |  |  |  |  |  |  |  |  |  |  |
| 59 |  |  |  |  |  |  |  |  |  |  |  |  |  |  |  |  |  |  |  |  |  |  |
| 60 |  |  |  |  |  |  |  |  |  |  |  |  |  |  |  |  |  |  |  |  |  |  |
| 61 |  |  |  |  |  |  |  |  |  |  |  |  |  |  |  |  |  |  |  |  |  |  |
| 62 |  |  |  |  |  |  |  |  |  |  |  |  |  |  |  |  |  |  |  |  |  |  |
| 63 |  |  |  |  |  |  |  |  |  |  |  |  |  |  |  |  |  |  |  |  |  |  |
| 64 |  |  |  |  |  |  |  |  |  |  |  |  |  |  |  |  |  |  |  |  |  |  |
| 65 |  |  |  |  |  |  |  |  |  |  |  |  |  |  |  |  |  |  |  |  |  |  |
| 66 |  |  |  |  |  |  |  |  |  |  |  |  |  |  |  |  |  |  |  |  |  |  |
| 67 |  |  |  |  |  |  |  |  |  |  |  |  |  |  |  |  |  |  |  |  |  |  |
| 68 |  |  |  |  |  |  |  |  |  |  |  |  |  |  |  |  |  |  |  |  |  |  |
| 69 |  |  |  |  |  |  |  |  |  |  |  |  |  |  |  |  |  |  |  |  |  |  |
| 70 |  |  |  |  |  |  |  |  |  |  |  |  |  |  |  |  |  |  |  |  |  |  |
| 71 |  |  |  |  |  |  |  |  |  |  |  |  |  |  |  |  |  |  |  |  |  |  |
| 72 |  |  |  |  |  |  |  |  |  |  |  |  |  |  |  |  |  |  |  |  |  |  |
| 73 |  |  |  |  |  |  |  |  |  |  |  |  |  |  |  |  |  |  |  |  |  |  |
| 74 |  |  |  |  |  |  |  |  |  |  |  |  |  |  |  |  |  |  |  |  |  |  |
| 75 |  |  |  |  |  |  |  |  |  |  |  |  |  |  |  |  |  |  |  |  |  |  |
| 76 |  |  |  |  |  |  |  |  |  |  |  |  |  |  |  |  |  |  |  |  |  |  |
| 77 |  |  |  |  |  |  |  |  |  |  |  |  |  |  |  |  |  |  |  |  |  |  |
| 78 |  |  |  |  |  |  |  |  |  |  |  |  |  |  |  |  |  |  |  |  |  |  |
| 79 |  |  |  |  |  |  |  |  |  |  |  |  |  |  |  |  |  |  |  |  |  |  |
| 80 |  |  |  |  |  |  |  |  |  |  |  |  |  |  |  |  |  |  |  |  |  |  |
| 81 |  |  |  |  |  |  |  |  |  |  |  |  |  |  |  |  |  |  |  |  |  |  |
| 82 |  |  |  |  |  |  |  |  |  |  |  |  |  |  |  |  |  |  |  |  |  |  |
| 83 |  |  |  |  |  |  |  |  |  |  |  |  |  |  |  |  |  |  |  |  |  |  |
| 84 |  |  |  |  |  |  |  |  |  |  |  |  |  |  |  |  |  |  |  |  |  |  |
| 85 |  |  |  |  |  |  |  |  |  |  |  |  |  |  |  |  |  |  |  |  |  |  |
| 86 |  |  |  |  |  |  |  |  |  |  |  |  |  |  |  |  |  |  |  |  |  |  |
| 87 |  |  |  |  |  |  |  |  |  |  |  |  |  |  |  |  |  |  |  |  |  |  |
| 88 |  |  |  |  |  |  |  |  |  |  |  |  |  |  |  |  |  |  |  |  |  |  |
| 89 |  |  |  |  |  |  |  |  |  |  |  |  |  |  |  |  |  |  |  |  |  |  |
| 90 |  |  |  |  |  |  |  |  |  |  |  |  |  |  |  |  |  |  |  |  |  |  |
| 91 |  |  |  |  |  |  |  |  |  |  |  |  |  |  |  |  |  |  |  |  |  |  |
| 92 |  |  |  |  |  |  |  |  |  |  |  |  |  |  |  |  |  |  |  |  |  |  |
| 93 |  |  |  |  |  |  |  |  |  |  |  |  |  |  |  |  |  |  |  |  |  |  |
| 94 |  |  |  |  |  |  |  |  |  |  |  |  |  |  |  |  |  |  |  |  |  |  |
| 95 |  |  |  |  |  |  |  |  |  |  |  |  |  |  |  |  |  |  |  |  |  |  |
| 96 |  |  |  |  |  |  |  |  |  |  |  |  |  |  |  |  |  |  |  |  |  |  |
| 97 |  |  |  |  |  |  |  |  |  |  |  |  |  |  |  |  |  |  |  |  |  |  |
| 98 |  |  |  |  |  |  |  |  |  |  |  |  |  |  |  |  |  |  |  |  |  |  |
| 99 |  |  |  |  |  |  |  |  |  |  |  |  |  |  |  |  |  |  |  |  |  |  |
| 100 |  |  |  |  |  |  |  |  |  |  |  |  |  |  |  |  |  |  |  |  |  |  |
| 101 |  |  |  |  |  |  |  |  |  |  |  |  |  |  |  |  |  |  |  |  |  |  |
| 102 |  |  |  |  |  |  |  |  |  |  |  |  |  |  |  |  |  |  |  |  |  |  |
| 103 |  |  |  |  |  |  |  |  |  |  |  |  |  |  |  |  |  |  |  |  |  |  |
| 104 |  |  |  |  |  |  |  |  |  |  |  |  |  |  |  |  |  |  |  |  |  |  |
| 105 |  |  |  |  |  |  |  |  |  |  |  |  |  |  |  |  |  |  |  |  |  |  |
| 106 |  |  |  |  |  |  |  |  |  |  |  |  |  |  |  |  |  |  |  |  |  |  |
| 107 |  |  |  |  |  |  |  |  |  |  |  |  |  |  |  |  |  |  |  |  |  |  |
| 108 |  |  |  |  |  |  |  |  |  |  |  |  |  |  |  |  |  |  |  |  |  |  |
| 109 |  |  |  |  |  |  |  |  |  |  |  |  |  |  |  |  |  |  |  |  |  |  |
| 110 |  |  |  |  |  |  |  |  |  |  |  |  |  |  |  |  |  |  |  |  |  |  |
| 111 |  |  |  |  |  |  |  |  |  |  |  |  |  |  |  |  |  |  |  |  |  |  |
| 112 |  |  |  |  |  |  |  |  |  |  |  |  |  |  |  |  |  |  |  |  |  |  |
| 113 |  |  |  |  |  |  |  |  |  |  |  |  |  |  |  |  |  |  |  |  |  |  |
| 114 |  |  |  |  |  |  |  |  |  |  |  |  |  |  |  |  |  |  |  |  |  |  |
| 115 |  |  |  |  |  |  |  |  |  |  |  |  |  |  |  |  |  |  |  |  |  |  |
| 116 |  |  |  |  |  |  |  |  |  |  |  |  |  |  |  |  |  |  |  |  |  |  |
| 117 |  |  |  |  |  |  |  |  |  |  |  |  |  |  |  |  |  |  |  |  |  |  |
| 118 |  |  |  |  |  |  |  |  |  |  |  |  |  |  |  |  |  |  |  |  |  |  |
| 119 |  |  |  |  |  |  |  |  |  |  |  |  |  |  |  |  |  |  |  |  |  |  |
| 120 |  |  |  |  |  |  |  |  |  |  |  |  |  |  |  |  |  |  |  |  |  |  |
| 121 |  |  |  |  |  |  |  |  |  |  |  |  |  |  |  |  |  |  |  |  |  |  |
| 122 |  |  |  |  |  |  |  |  |  |  |  |  |  |  |  |  |  |  |  |  |  |  |
| 123 |  |  |  |  |  |  |  |  |  |  |  |  |  |  |  |  |  |  |  |  |  |  |
| 124 |  |  |  |  |  |  |  |  |  |  |  |  |  |  |  |  |  |  |  |  |  |  |
| 125 |  |  |  |  |  |  |  |  |  |  |  |  |  |  |  |  |  |  |  |  |  |  |
| 126 |  |  |  |  |  |  |  |  |  |  |  |  |  |  |  |  |  |  |  |  |  |  |
| 127 |  |  |  |  |  |  |  |  |  |  |  |  |  |  |  |  |  |  |  |  |  |  |
| 128 |  |  |  |  |  |  |  |  |  |  |  |  |  |  |  |  |  |  |  |  |  |  |
| 129 |  |  |  |  |  |  |  |  |  |  |  |  |  |  |  |  |  |  |  |  |  |  |
| 130 |  |  |  |  |  |  |  |  |  |  |  |  |  |  |  |  |  |  |  |  |  |  |
| 131 |  |  |  |  |  |  |  |  |  |  |  |  |  |  |  |  |  |  |  |  |  |  |
| 132 |  |  |  |  |  |  |  |  |  |  |  |  |  |  |  |  |  |  |  |  |  |  |
| 133 |  |  |  |  |  |  |  |  |  |  |  |  |  |  |  |  |  |  |  |  |  |  |
| 134 |  |  |  |  |  |  |  |  |  |  |  |  |  |  |  |  |  |  |  |  |  |  |
| 135 |  |  |  |  |  |  |  |  |  |  |  |  |  |  |  |  |  |  |  |  |  |  |
| 136 |  |  |  |  |  |  |  |  |  |  |  |  |  |  |  |  |  |  |  |  |  |  |
| 137 |  |  |  |  |  |  |  |  |  |  |  |  |  |  |  |  |  |  |  |  |  |  |
| 138 |  |  |  |  |  |  |  |  |  |  |  |  |  |  |  |  |  |  |  |  |  |  |
| 139 |  |  |  |  |  |  |  |  |  |  |  |  |  |  |  |  |  |  |  |  |  |  |
| 140 |  |  |  |  |  |  |  |  |  |  |  |  |  |  |  |  |  |  |  |  |  |  |
| 141 |  |  |  |  |  |  |  |  |  |  |  |  |  |  |  |  |  |  |  |  |  |  |
| 142 |  |  |  |  |  |  |  |  |  |  |  |  |  |  |  |  |  |  |  |  |  |  |
| 143 |  |  |  |  |  |  |  |  |  |  |  |  |  |  |  |  |  |  |  |  |  |  |
| 144 |  |  |  |  |  |  |  |  |  |  |  |  |  |  |  |  |  |  |  |  |  |  |
| 145 |  |  |  |  |  |  |  |  |  |  |  |  |  |  |  |  |  |  |  |  |  |  |
| 146 |  |  |  |  |  |  |  |  |  |  |  |  |  |  |  |  |  |  |  |  |  |  |
| 147 |  |  |  |  |  |  |  |  |  |  |  |  |  |  |  |  |  |  |  |  |  |  |
| 148 |  |  |  |  |  |  |  |  |  |  |  |  |  |  |  |  |  |  |  |  |  |  |
| 149 |  |  |  |  |  |  |  |  |  |  |  |  |  |  |  |  |  |  |  |  |  |  |
| 150 |  |  |  |  |  |  |  |  |  |  |  |  |  |  |  |  |  |  |  |  |  |  |
| 151 |  |  |  |  |  |  |  |  |  |  |  |  |  |  |  |  |  |  |  |  |  |  |
| 152 |  |  |  |  |  |  |  |  |  |  |  |  |  |  |  |  |  |  |  |  |  |  |
| 153 |  |  |  |  |  |  |  |  |  |  |  |  |  |  |  |  |  |  |  |  |  |  |
| 154 |  |  |  |  |  |  |  |  |  |  |  |  |  |  |  |  |  |  |  |  |  |  |
| 155 |  |  |  |  |  |  |  |  |  |  |  |  |  |  |  |  |  |  |  |  |  |  |
| 156 |  |  |  |  |  |  |  |  |  |  |  |  |  |  |  |  |  |  |  |  |  |  |
| 157 |  |  |  |  |  |  |  |  |  |  |  |  |  |  |  |  |  |  |  |  |  |  |
| 158 |  |  |  |  |  |  |  |  |  |  |  |  |  |  |  |  |  |  |  |  |  |  |
| 159 |  |  |  |  |  |  |  |  |  |  |  |  |  |  |  |  |  |  |  |  |  |  |
| 160 |  |  |  |  |  |  |  |  |  |  |  |  |  |  |  |  |  |  |  |  |  |  |
| 161 |  |  |  |  |  |  |  |  |  |  |  |  |  |  |  |  |  |  |  |  |  |  |
| 162 |  |  |  |  |  |  |  |  |  |  |  |  |  |  |  |  |  |  |  |  |  |  |
| 163 |  |  |  |  |  |  |  |  |  |  |  |  |  |  |  |  |  |  |  |  |  |  |

**APPENDIX 2 – MOHAWk data summary form**


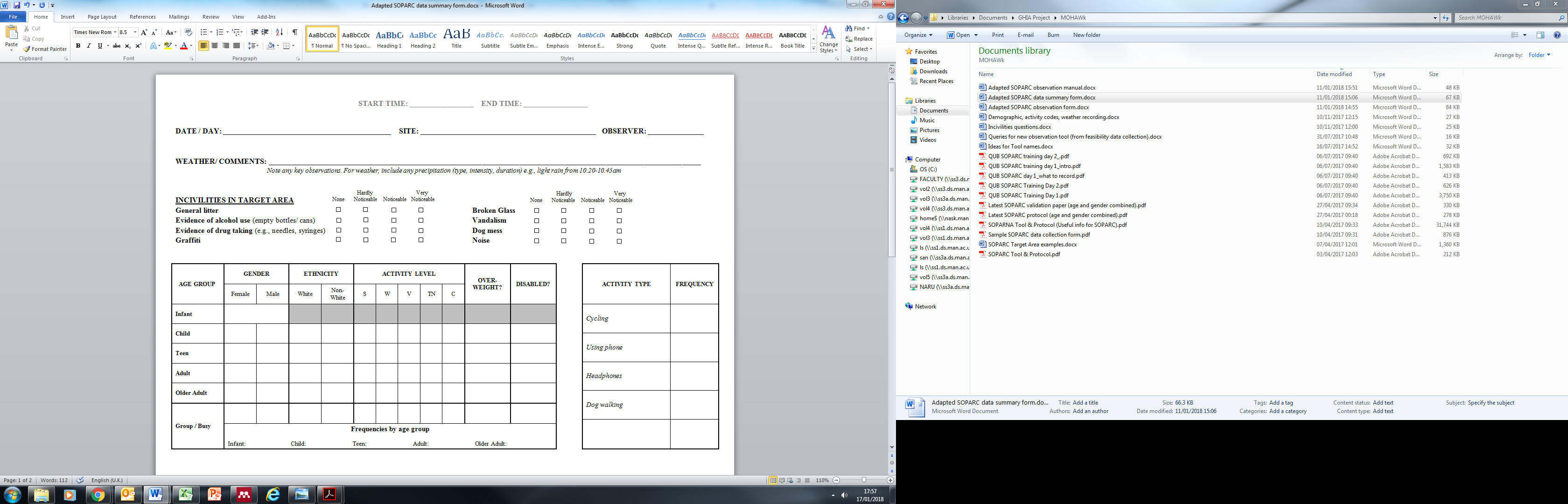


**APPENDIX 3 – Example of a completed MOHAWK observation form**


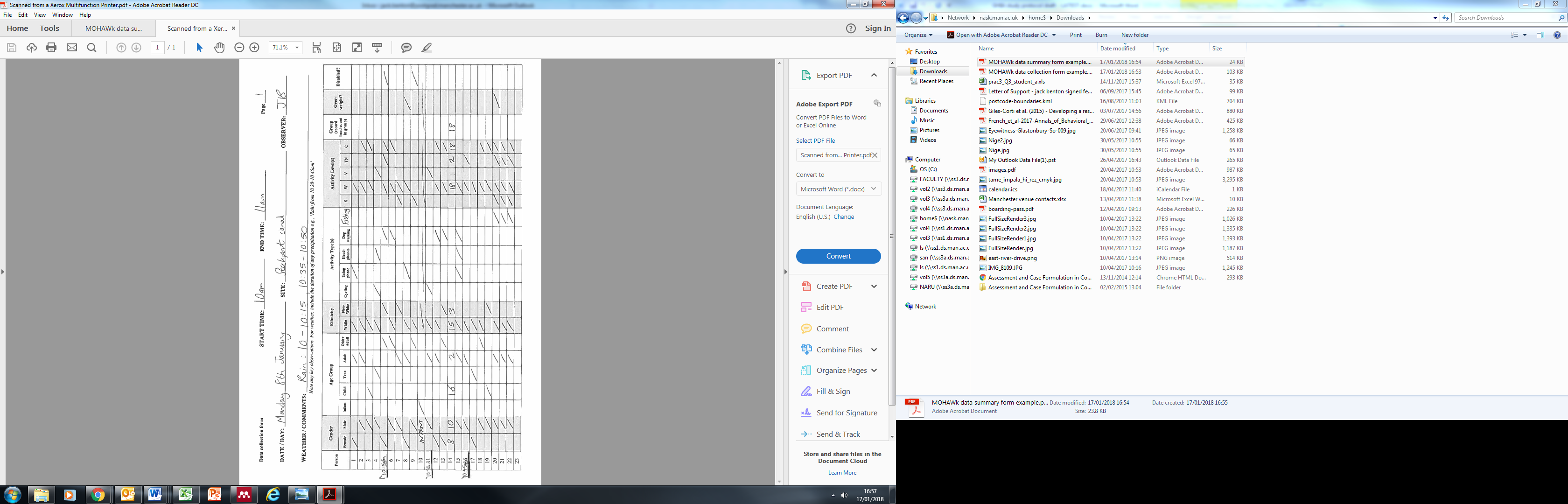


**APPENDIX 4 – Example of a completed MOHAWK data summary form**


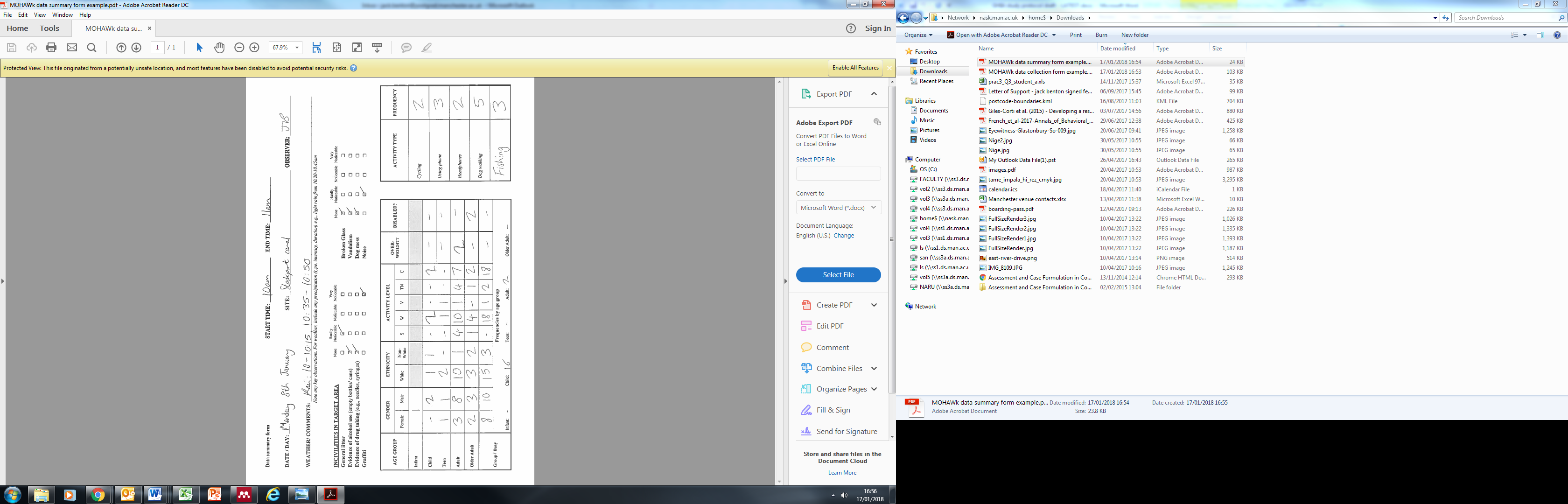


1. This is a preliminary version of the Description and Procedures Manual for Method for Observing Physical Activity and Wellbeing (MOHAWk). Validation studies for the MOHAWk are still ongoing and the finalised version of this manual will be published once the validation studies are completed and published. [↑](#footnote-ref-1)
2. McKenzie TL, Cohen DA, Sehgal A, Williamson S, Golinelli D. System for Observing Play and Recreation in Communities (SOPARC): reliability and feasibility measures. J Phys Act Health. 2006;3(s1):S208-22. [↑](#footnote-ref-2)
