# Supplementary material for: Evaluating the impact of improvements in urban green space on older adults’ physical activity and wellbeing: protocol for a natural experimental study
Source: BMC Public Health. 2018 Jul 27;18:923. doi: 10.1186/s12889-018-5812-z (PMC6062989; doi:10.1186/s12889-018-5812-z)

**Target Area boundaries for each intervention and comparison site**

This document provides maps of the boundaries for the Target Areas in each intervention and comparison site. Each map also shows the primary location for the observer during observation periods and locations where the urban street greening interventions will be implemented at each intervention site.

**Key**

**X** = Observer location


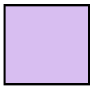


= Target area boundary


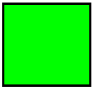


= Location where the urban street greening interventions will be implemented

Maps drawn using: www.digimap.edina.ac.uk

© Crown Copyright and Database Right (2018). Ordnance Survey (Digimap Licence)

**Intervention site 1**


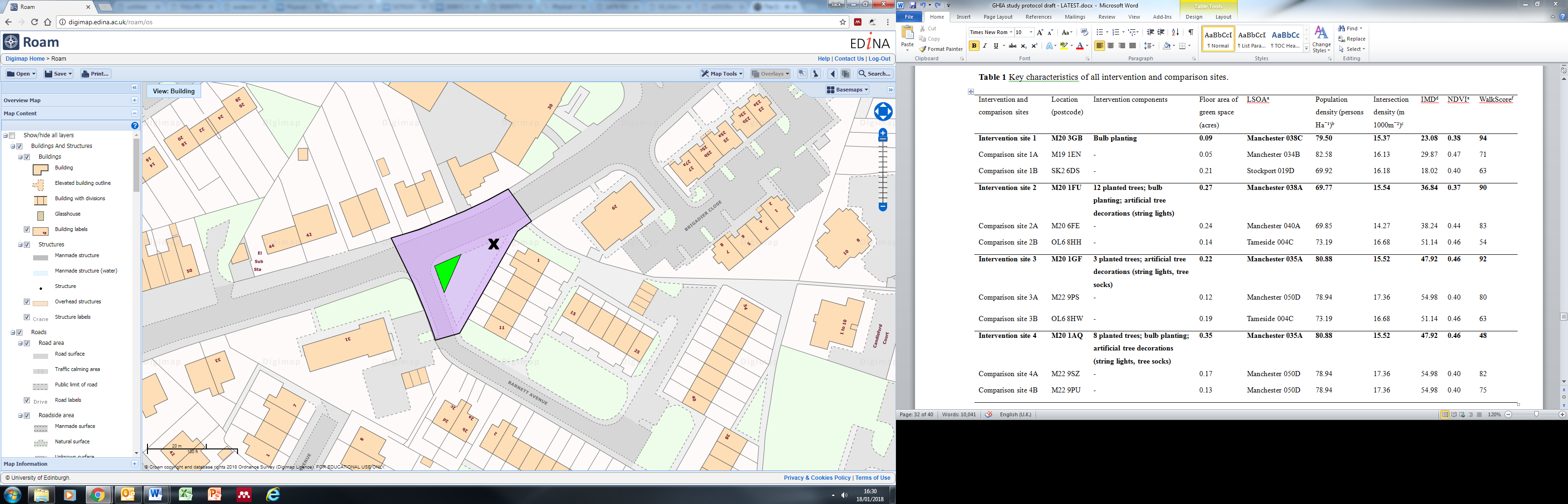


**Comparison site 1A**


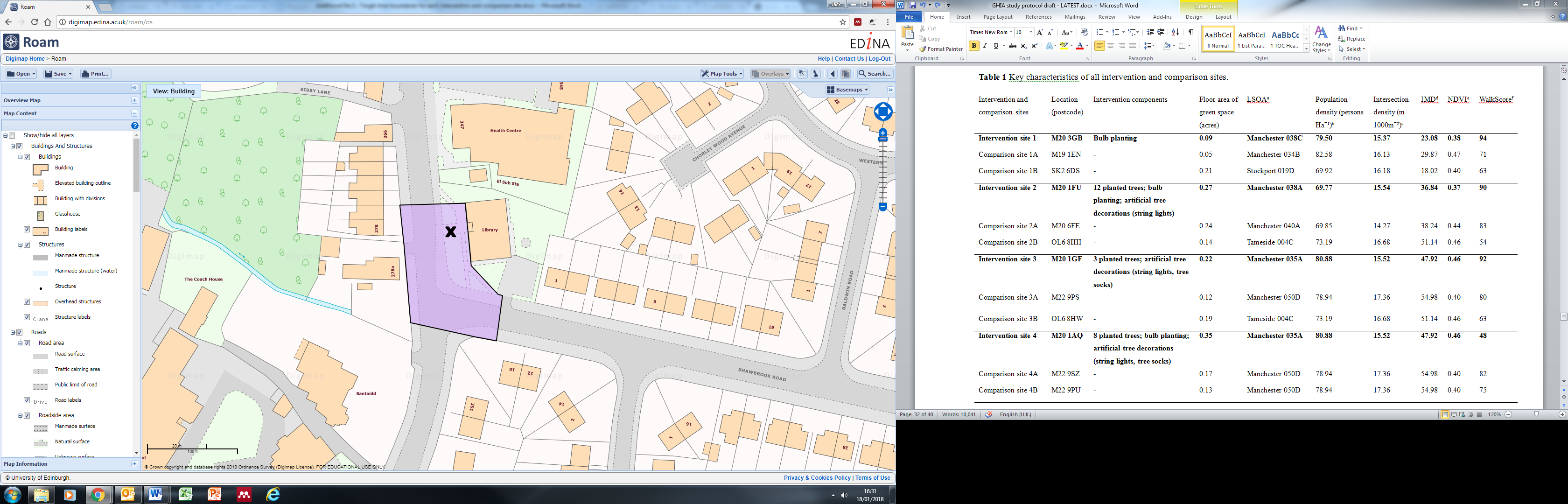


**Comparison site 1B**


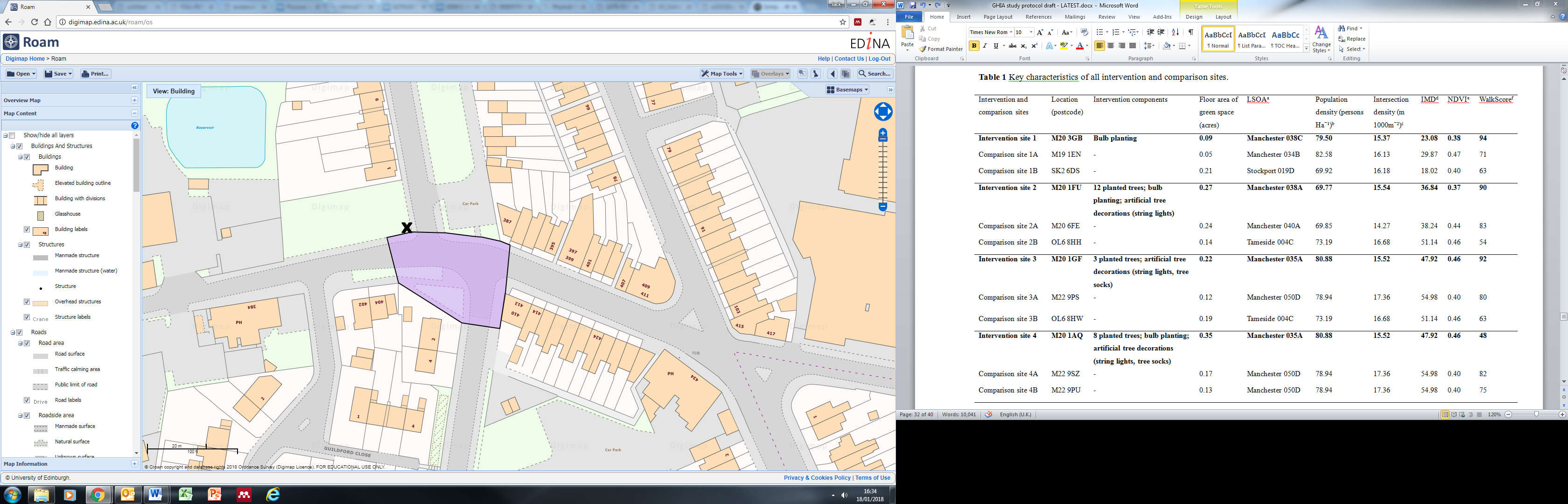


**Intervention site 2**


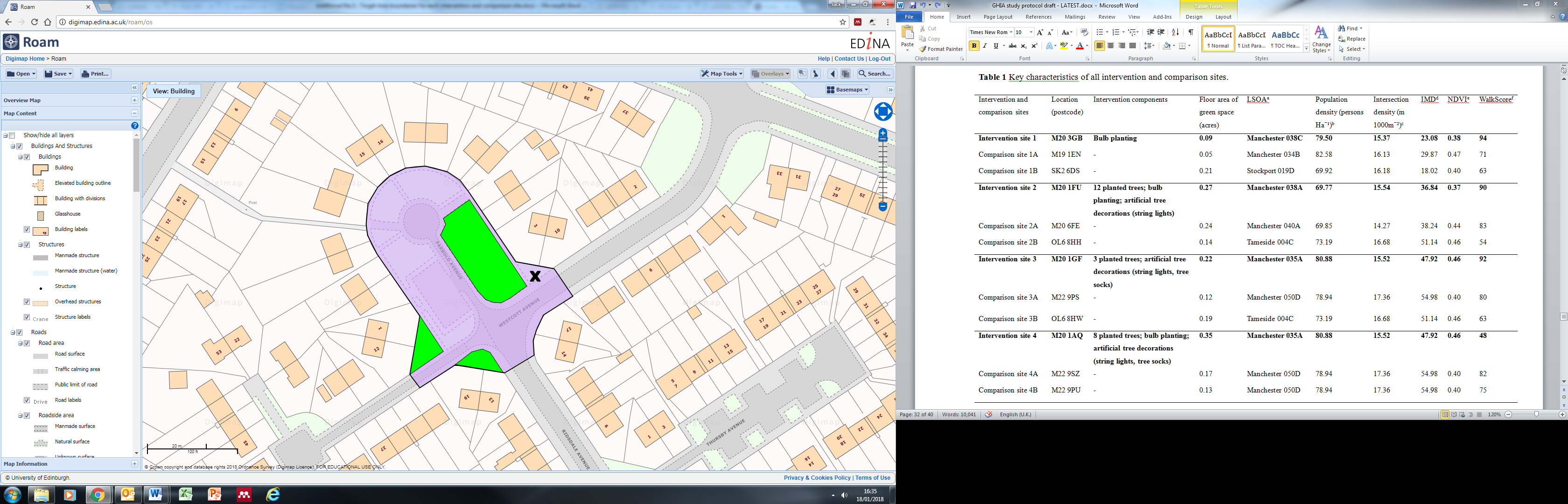


**Comparison site 2A**


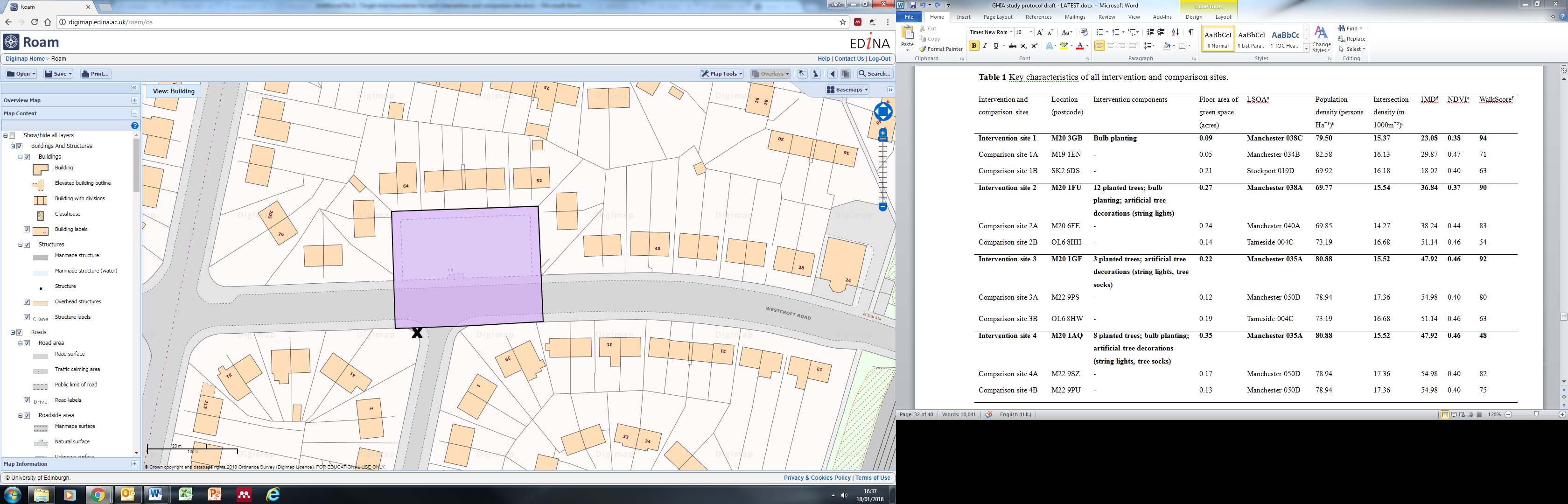


**Comparison site 2B**


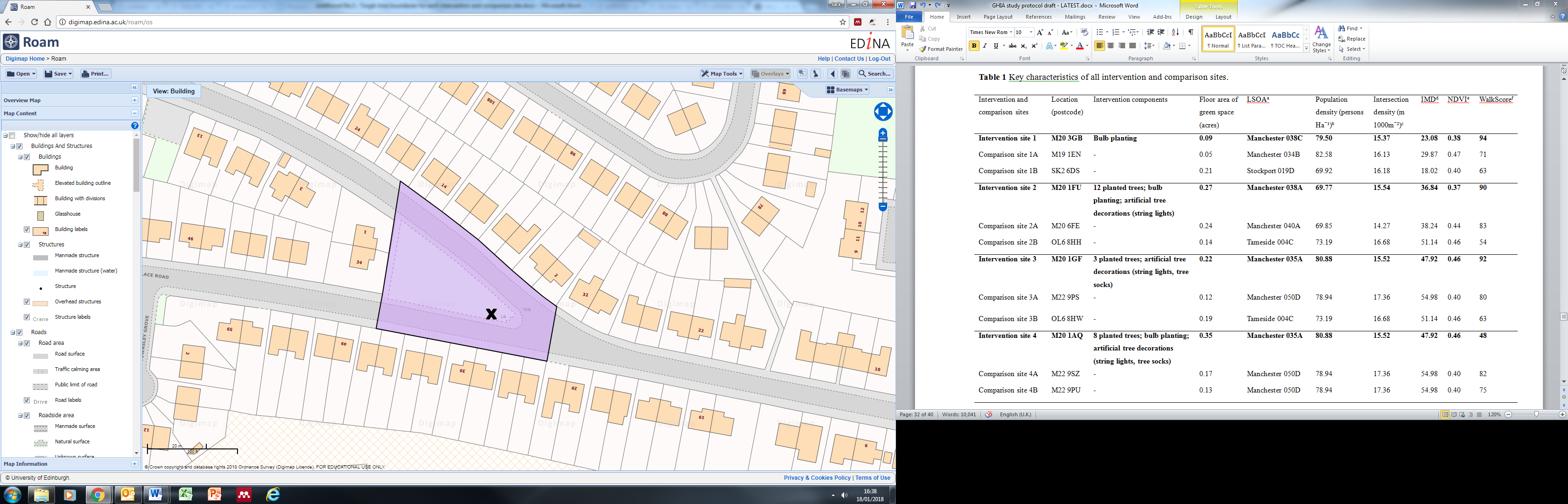


**Intervention site 3**


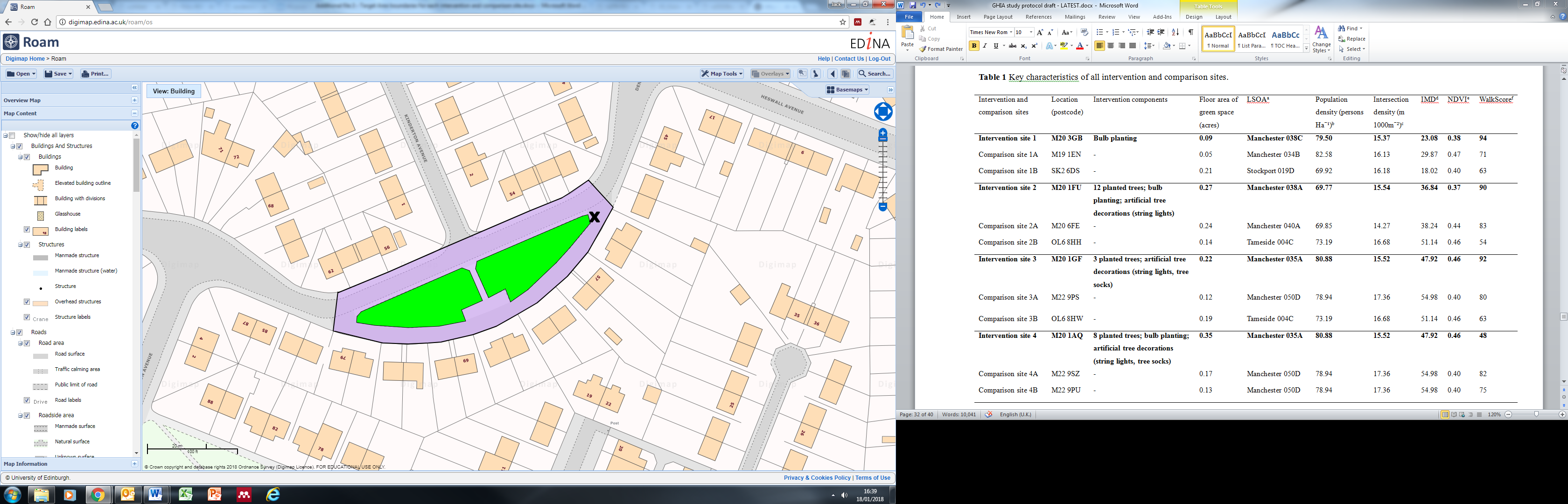


**Comparison site 3A**


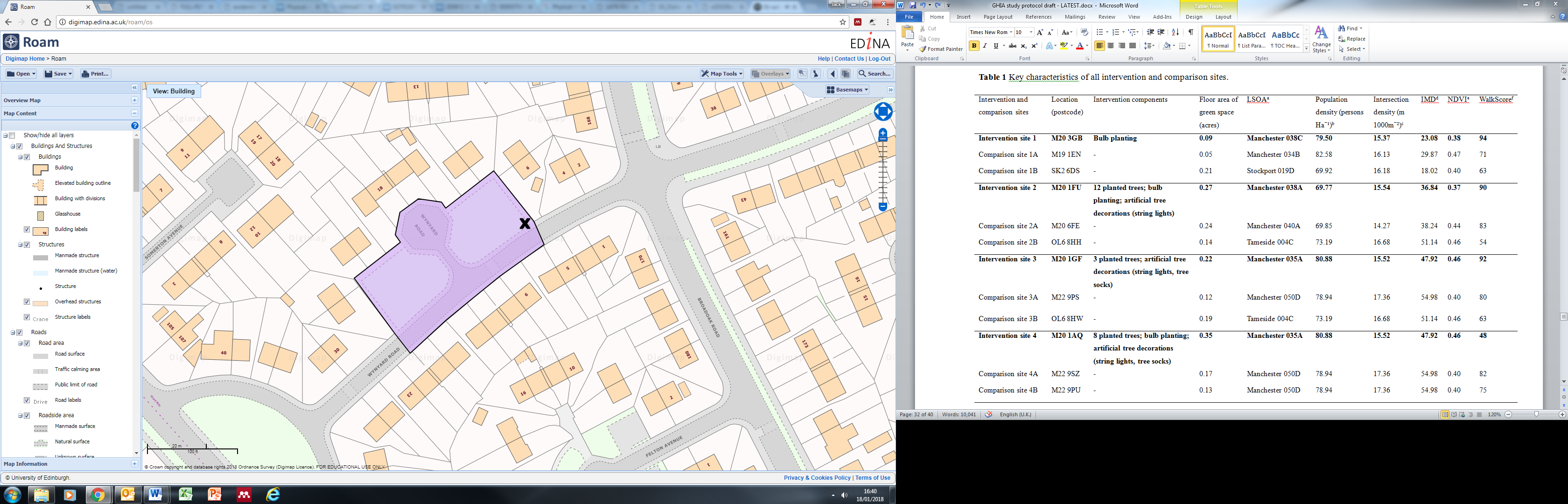


**Comparison site 3B**


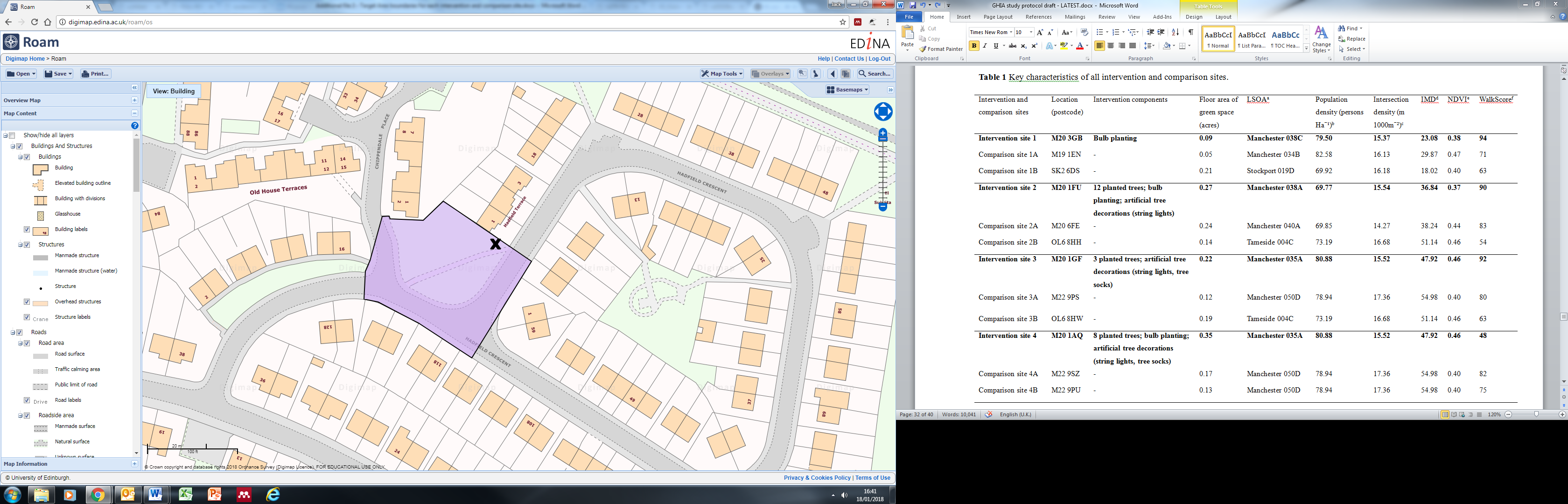


**Intervention site 4**


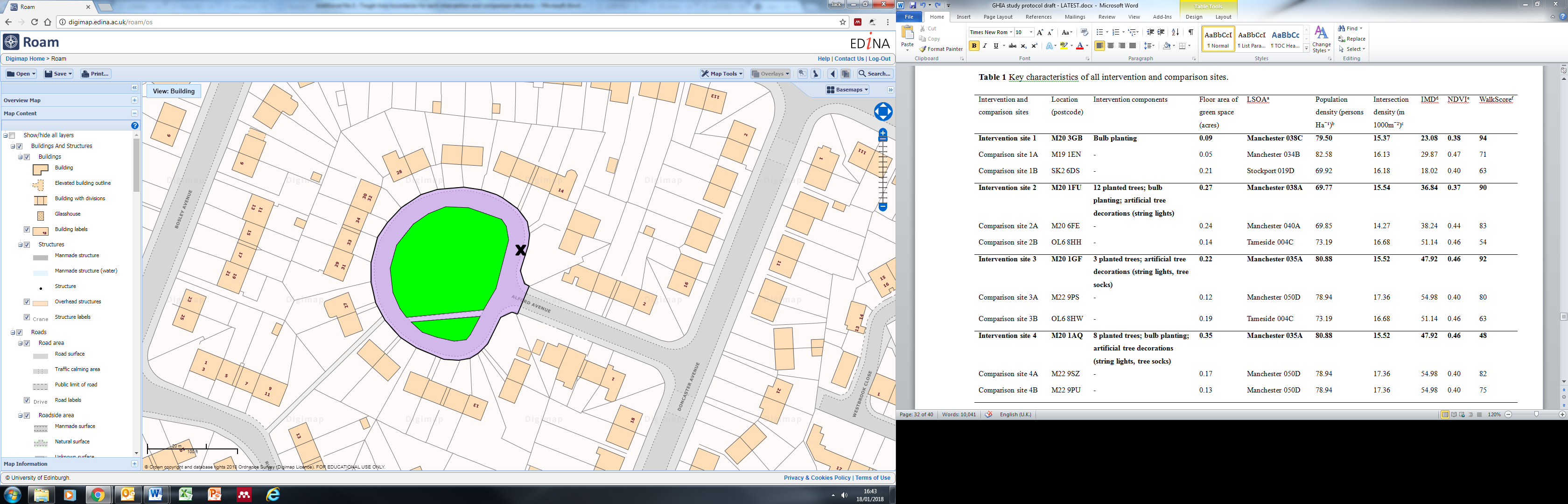


**Comparison site 4A**


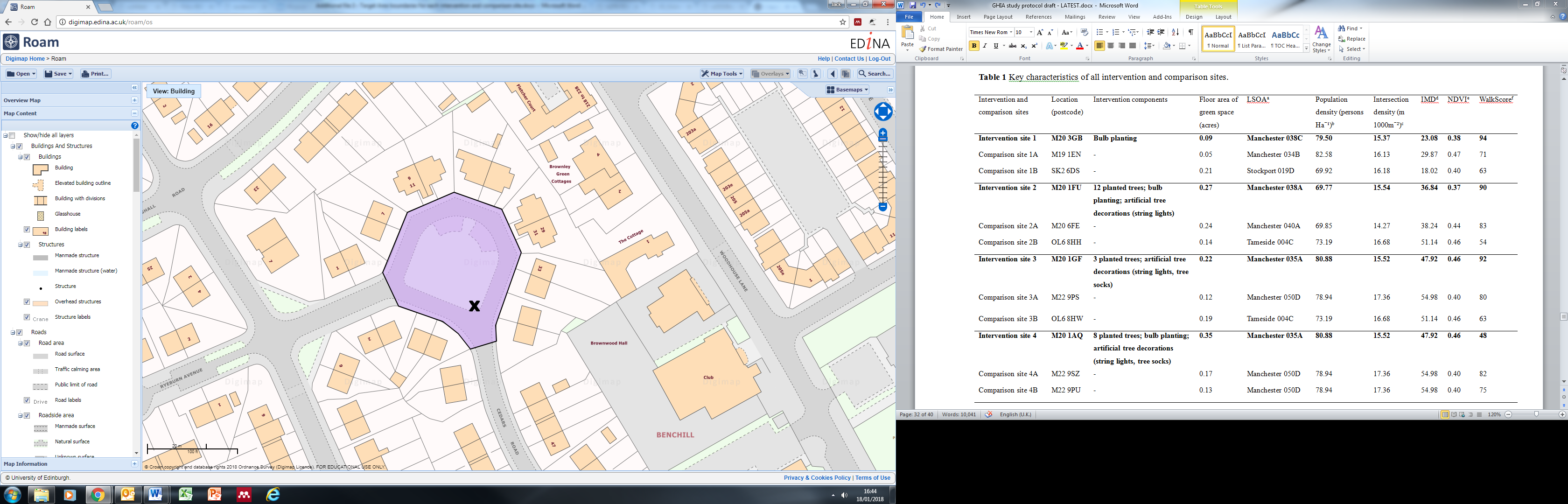


**Comparison site 4B**


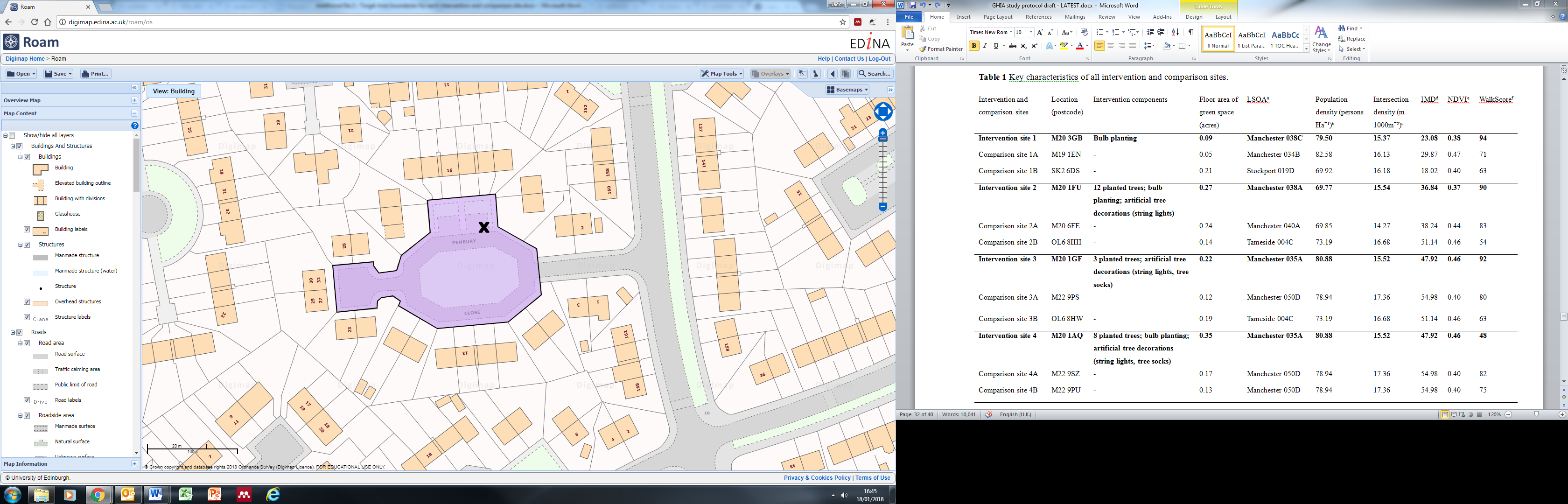

Supplement: Supplementary file 3 — Target area boundaries for each intervention and comparison site. (DOCX 10549 kb) [file 12889_2018_5812_MOESM3_ESM.docx]
